# The Impact of Vitamin D Supplementation on Fasting Plasma Glucose, Insulin Sensitivity, and Inflammation in Type 2 Diabetes Mellitus: A Systematic Review and Meta-Analysis

**DOI:** 10.3390/nu17152489

**Published:** 2025-07-30

**Authors:** Enny Probosari, Hertanto W. Subagio, Heri-Nugroho, Banundari Rachmawati, Siti F. Muis, Kevin C. Tjandra, Dwi Adiningsih, Tri I. Winarni

**Affiliations:** 1 Doctoral Program of Medical and Health Sciences, Faculty of Medicine, Universitas Diponegoro, Semarang 50275, Central Java, Indonesia; 2 Clinical Nutrition Department, Faculty of Medicine, Universitas Diponegoro, Semarang 50275, Central Java, Indonesia; 3 Division of Endocrinology, Metabolism and Diabetes, Department of Internal Medicine, Faculty of Medicine, Universitas Diponegoro-Dr. Kariadi General Hospital, Semarang 50275, Central Java, Indonesia; 4 Department of Clinical Pathology, Faculty of Medicine, Universitas Diponegoro-Dr. Kariadi General Hospital, Semarang 50275, Central Java, Indonesia; 5 Department of Surgery, Faculty of Medicine, Universitas Diponegoro-Dr. Kariadi General Hospital, Semarang 50275, Central Java, Indonesia; 6 Department of Undergraduate, Faculty of Medicine, Universitas Diponegoro-Dr. Kariadi General Hospital, Semarang 50275, Central Java, Indonesia; 7 Department of Anatomy, Faculty of Medicine, Universitas Diponegoro, Semarang 50275, Central Java, Indonesia; 8 Undip Biomechanics Engineering & Research Centre (UBM-ERC), Universitas Diponegoro, Semarang 50275, Central Java, Indonesia

**Keywords:** biomarkers, fasting plasma glucose, hs-CRP, insulin sensitivity, type 2 diabetes mellitus, vitamin D

## Abstract

**Background/Objectives**: Vitamin D supplementation has shown promise in managing type 2 diabetes mellitus (T2DM), while the simultaneous impact on glycemic control and inflammation in T2DM remains poorly understood. This study aimed to investigate the potential role of vitamin D supplementation in managing T2DM using fasting plasma glucose (FPG), insulin levels, HOMA-IR, HOMA-B, HbA1c, and Hs-CRP as the biomarkers. **Methods**: Original articles from Scopus, Pubmed, Cochrane Library, Epistemonikos, and ScienceDirect published between 2014 and 2024 were the sources. Inclusion criteria included studies conducted as clinical trials or randomized controlled trials involving adult patients diagnosed with T2DM undergoing treatment with vitamin D. The risk of bias was evaluated using the ROB-2 tool and meta-analysis was conducted to quantitatively synthesize the results across the studies using pooled effect sizes and confidence intervals. **Results**: Nine studies were included in the meta-analysis. Significant differences were found at 12-week follow-up in insulin level (MD(−3.59) [95% CI: −6.93, −0.25]), HOMA-B (MD(−50.35) [95% CI: −92.29, −8.41]), hs-CRP (−2.51 [95% CI: −3.45, −1.57]), and HbA1c level (MD(−0.30) [95% CI: −0.54, −0.06]) and at 24-week follow-up in HOMA-IR (MD(−0.38) [CI: −0.53, −0.24]). The quality of the included studies was generally moderate, with three showing a potential risk of bias. **Conclusions**: The observed trends in FPG, insulin levels, HOMA-IR, HOMA-B, HbA1c, and hs-CRP indicate that vitamin D may influence glycemic control, insulin sensitivity, and inflammation, but these effects are often modest and may diminish over time. Future studies should explore longer duration randomized trials with standardized dosing and baseline vitamin D status stratification.

## 1. Introduction

Vitamin D is an essential micronutrient that influences human health by modulating immunity, cell growth, inflammation, and genomic processes affecting cell death, differentiation, and proliferation [1,2]. Calcitriol (1,25(OH)2D3), the active form of vitamin D, is formed by the conversion of ergocalciferol (vitamin D2) and cholecalciferol (vitamin D3), which undergoes hepatic and renal metabolism [2]. Vitamin D deficiency is linked to metabolic syndrome, obesity, and type 2 diabetes. It plays a role in regulating insulin secretion and sensitivity, which are vital for metabolic health [3]. In a cross-sectional study of 89 overweight and obese adults, vitamin D deficiency was highly prevalent (93.2%) and a significant positive correlation was observed between vitamin D levels and fasting blood sugar [4]. In an observational study of 110 elderly Caucasian patients (>60 years), vitamin D deficiency was highly prevalent (84.5%) and significantly associated with visceral obesity, suggesting a potential role of vitamin D in managing obesity in the elderly [5].

Type 2 diabetes mellitus (T2DM) prevalence is increasing globally, reaching 10.5% in the last three decades [6]. T2DM is a chronic metabolic disorder marked by persistently high blood sugar levels brought on by insulin resistance, impaired insulin secretion, or both [7,8]. It has long been known that glucose intolerance is linked to hypovitaminosis D [9]. Individuals who were at risk for diabetes had lower levels of 25(OH)D than those who were not. Hypovitaminosis D has been linked to decreased insulin secretion in a diabetic population [10]. In older males with hypovitaminosis D, insulin secretion is hyperresponsive to a glucose challenge. Thus, throughout the development of T2DM, vitamin D may have an impact on insulin sensitivity and beta cell function, as well as on both [11].

Vitamin D corrects vitamin D deficiency in T2DM by supporting and modulating insulin production and secretion through several mechanisms. One of them is by the binding of the active form of vitamin D, 1,25(OH)D with VDR. This binding will induce glucose transport and insulin secretion through the cellular growth in β cells [12,13]. Vitamin D may also have the potential to regulate insulin creation in an indirect way by affecting the concentrations of intracellular calcium. Calcium triggers the release of insulin. 1,25(OH)_2_D can depolarize cytoplasmic membranes in β cells, which will open Ca^2+^ channels and increase intracellular Ca^2+^ levels [14,15]. This action may be triggered by activating PKA, enhancing the channel function by phosphorylating L-type voltage-dependent Ca^2+^ channel-related proteins, and by regulating the expression of voltage-gated calcium channels to increase the release of insulin [16,17]. Additionally, vitamin D promotes phospholipase C synthesis and activates inositol triphosphate that releases Ca^2+^ from the endoplasmic reticulum [18].

A number of meta-analyses have demonstrated significant positive outcomes of vitamin D supplementation on blood glucose. One study showed that vitamin D supplementation can significantly reduce serum fasting plasma glucose, HbA1c, HOMA-IR, and insulin levels in T2DM patients. However, that study did not investigate the durations and dosages of vitamin D supplements in the inflammation marker outcome in T2DM [19]. One study showed that vitamin D may reduce chronic low-grade inflammation in patients with T2DM, but did not consider the differences in outcomes based on the duration of supplementation [20].

Although a number of meta-analyses have demonstrated significant positive outcomes of vitamin D supplementation in T2DM, few have simultaneously assessed both glycemic and inflammatory markers within a single comprehensive evaluation, nor have they systematically explored the effects of intervention duration and dosage, particularly for inflammation outcomes. The novelty of the current study lies in its approach to analyzing both metabolic and inflammatory effects of vitamin D supplementation, while also incorporating a detailed risk of bias assessment and subgroup analyses based on intervention duration. This review aims to provide the most current and detailed qualitative summary of the evidence. Therefore, the aim of this systematic review and meta-analysis was to evaluate the impacts of vitamin D on both glycemic control and inflammation in adults with T2DM focusing on key biomarkers including fasting plasma glucose (FPG), insulin levels, HOMA-IR, HOMA-B, HbA1c, TNF-alpha, and Hs-CRP.

## 2. Materials and Methods

### 2.1. Study Design and Registration

This review complies with the Cochraine Handbook and the Preferred Reporting Items for Systematic Reviews and Meta-Analyses (PRISMA) statement [21,22]. The PRISMA checklist as used for our guidance is available in Supplementary File S1. This systematic review and meta-analysis were registered in OSF with registry at https://doi.org/10.17605/OSF.IO/4NFMH (accessed on 22 July 2024).

### 2.2. Eligibility Criteria

The following standards were used in the selection of the featured studies: (1) studies involving patients of adults diagnosed with T2DM undergoing treatment with vitamin D3; (2) comparisons of the treatment indicated as placebo; (3) availability of data on the primary outcome that consist of FPG, insulin level, HOMA-IR, HOMA-B, HbA1C, and Hs-CRP; and (4) studies conducted as a clinical trial or randomized control trials (RCTs). Conversely, we excluded studies that involved patients with metabolic diseases other than T2DM. Exclusion criteria included non-full text, scientific posters, study protocols, conference abstracts, non-English articles, irrelevant articles, other technical reports, systematic reviews, non-comparative research, narrative reviews, meta-analyses, editor answers, and in silico, in vitro, and in vivo studies.

### 2.3. Data Search and Selection

A selection of keywords was used to capture all pertinent studies for each database, as listed in below Table 1. The search was conducted from each database’s inception until 20 June 2025. The National Institutes of Health (NIH) National Library of Medicine browser’s definition of Medical Subject Headings (MeSH) keywords was subjected to the Boolean operator. Mendeley Group Reference Manager was used to arrange the references in the author’s library to organize full text screening and remove journal duplication. Three authors (EP, KCT, DA), who managed the literature search independently, performed the entire article selection process. Articles were first sorted by title and abstract. Duplicates were removed utilizing Mendeley Group Reference Manager. The remaining articles were thoroughly reviewed in full text by two authors (KCT and EP) to ascertain the eligibility based on our criteria for inclusion and exclusion. Any disagreements were resolved through discussions with the other authors (TIW, HN, HWS, BR, SFM), who provided a final assessment on the eligibility of studies for the synthesis. Furthermore, the manual screening through the reference list of all the included studies was also performed to help minimize the risk of missing studies.

### 2.4. Data Extraction

The first author’s last name, publication year, country, study design, sample size, baseline characteristics of participants (including mean age and sex distribution), follow-up duration, number of participants, and outcomes of interest were all retrieved for analytical purposes. Eight independent authors (E.P., T.I.W., H.N., H.W.S., B.R., S.F.M., K.C.T., D.A.) were responsible for extracting these data, which were then organized in Microsoft Excel 2019.

Only primary outcomes were considered in this study, which were fasting plasma glucose (FPG), insulin level, HOMA-IR, HOMA-B, HbA1C, TNF-alpha, TNF-alpha, and Hs-CRP, while our secondary outcome was the comparison level of vitamin D before and after intervention.

### 2.5. Risk of Bias Assessment

Five independent authors (EP, TIW, SFM, KCT, DA) used validated and firmly established tools to assess the risk of bias. This study utilized the Cochrane Collaboration’s Risk of Bias version 2 (RoB-2 tool) to evaluate five methodological domains in randomized controlled trials in this study: (a) the randomization procedure, (b) variations from the planned interventions, (c) absent data pertaining to outcomes, (d) outcome measurement, and (e) choosing the findings reported [23]. The results of this evaluation is shown in Supplementary File S3, which were classified as “low risk”, “high risk”, or having “some concerns” of bias. Disagreements during the assessment process were settled by discussion. The conclusion was determined by EP if no agreement could be reached.

### 2.6. Statistical Analysis

The inverse variance technique was utilized to collect the results of continuous variables using the standardized mean difference (SMD) with 95% confidence intervals (95% CIs). Because of the expected significant variability resulting from variations in follow-up times and demographics in the population, we chose to employ random-effect designs for this review [24].

The I-squared (I^2^) statistic was used to evaluate heterogeneity between studies. The heterogeneity value is classified as 0–30% not important, 30–50% moderate, 50–75% substantial, and >75% considerable [25]. Using an incorporated equation by Luo D et al. and Wan X et al., we turned values acknowledged as median and interquartile range (IQR) or median, minimum, and maximum to means and standard deviations (SDs) for pooled analysis.

The intervention and control groups were compared within follow-up period segments in our early analysis. In general, the findings indicated that the intervention group achieved greater functional results. Subgroup analyses and other studies, however, produced conflicting results, which led to a distinct analysis of prior to and after the procedure values. In order to compare the pooled means between the groups, this supplementary analysis sought to identify the mean difference for each evaluation irrespectively for both groups.

For any given result, provided there were more than ten studies, publication bias was examined. In cases where funnel plot asymmetry was observed, we examined the PICO and outcome elements to see if the problem was caused by publication bias or methodological differences between studies. The Cochrane Collaboration’s Review Manager 5.4 was used for all statistical analysis, with sensitivity meta-analysis assessing the robustness of outcomes through studies with a low risk of bias.

The certainty of evidence for each outcome was assessed using the Grading of Recommendations, Assessment, Development, and Evaluation (GRADE) approach. We evaluated five domains that could potentially downgrade the quality of evidence—risk of bias, inconsistency, indirectness, imprecision, and publication bias. Each domain was scored as having no limitation (0), serious limitation (−1), or very serious limitation (−2). The overall certainty of evidence was then classified as high, moderate, low, or very low based on the cumulative score. Since all the included studies were randomized controlled trials, each outcome initially started as high-quality evidence and was downgraded as necessary according to domain assessments. The scoring process was conducted independently by two reviewers, and any discrepancies were resolved through discussion. A summary table of the GRADE scoring is provided in the results section.

To assess the robustness of the pooled estimates and identify potential sources of heterogeneity, a leave-one-out sensitivity analysis was conducted. This method involves sequentially removing one study at a time from the meta-analysis and recalculating the overall effect estimate for each iteration. By observing the extent to which the pooled results change with the exclusion of individual studies, we were able to evaluate the influence of each study on the overall findings. Significant shifts in the effect size or confidence intervals upon exclusion of a particular study were interpreted as indicators of potential outlier influence or study-level bias. This analysis was performed using the “metainf” function from the meta package in R Studio Version 2024.12.1+563.

## 3. Results

### 3.1. Study Selection

**Figure 1:**
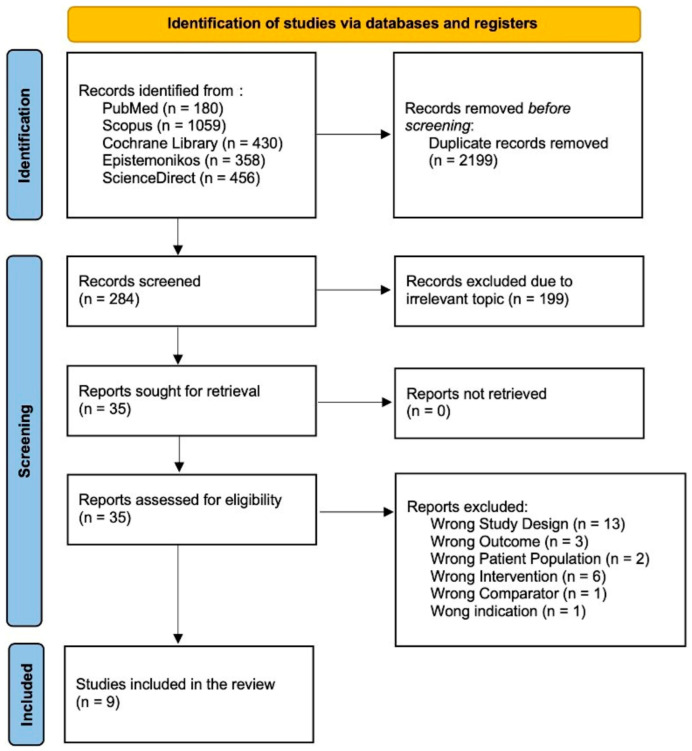
Flow diagram for PRISMA 2020.

A literature search conducted across PubMed, Scopus, Cochrane Library, Epistemonikos, and Science Direct initially yielded 1892 papers. Automation tools across each database were employed to filter out non-randomized controlled trial and non-clinical trial studies, resulting in the removal of 1658 articles. Subsequently, the authors manually screened the remaining articles for relevance and duplication, starting with titles and abstracts. This screening process led to the exclusion of 13 studies due to incorrect study design, 3 studies due to incorrect outcomes, 2 studies due to incorrect patient populations, 6 studies due to incorrect interventions, 1 study due to an incorrect comparator, and 1 study due to incorrect indications. After this rigorous selection process, the authors determined that nine studies met the inclusion criteria. Consequently, this systematic review and meta-analysis includes nine studies. The study screening report is available in Supplementary File S2, while Figure 1 shows the PRISMA diagram that represents our study selection process.

### 3.2. Risk of Bias in Studies

The quality of each clinical trial and randomized controlled trial included in this review was rigorously evaluated using the ROB-2 risk-of-bias tool. This method provides a comprehensive assessment of potential biases in several critical areas of study design. Notably, among the nine investigations analyzed, three studies raised concerns due to ambiguity in the randomization process, particularly in how participants were allocated to the intervention and control groups. This uncertainty may introduce a risk of bias, as the lack of clear randomization can affect the study’s internal validity. A detailed summary of the risk-of-bias assessment for all the included studies is presented in Supplementary File S3.

### 3.3. Study Result Summaries

The table presents the overview of nine randomized controlled trials (RCTs) conducted across several countries (Iran, India, Serbia, Iran, Netherlands, Australia, Mexico, and China), examining the effects of different vitamin D supplementation regimens on patient outcomes. The studies involved diverse populations, with sample sizes ranging from 30 to 261 participants, and supplementation doses varying from 1000 IU/day to 100,000 IU bolus followed by daily doses. Follow-up periods spanned from 12 weeks to 78 weeks, reflecting a broad spectrum of intervention durations. Most studies reported the mean ages of participants in both the supplementation and placebo groups, indicating comparable baseline characteristics across groups. Despite the geographical and dosage variations, these studies collectively contribute valuable insights into the potential benefits of vitamin D supplementation in different clinical contexts. The characteristics are shown in Table 2.

### 3.4. Fasting Plasma Glucose (FPG)

**Figure 2:**
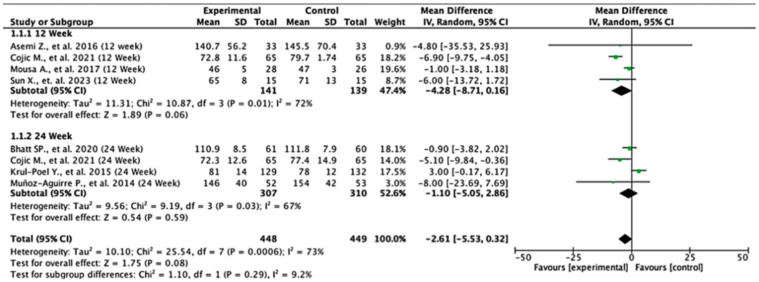
Forest plot for the FPG outcome. All studies shown are referenced as: Asemi Z., et al., (2016), Cojic M., et al., (2021), Mousa A., et al., (2017), Sun X., et al., (2023), Bhatt SP., et al., (2020), Krul-Poel Y., et al., (2015), Muñoz-Aguirre P., et al., (2014) [26,28,29,30,31,32,33].

Across the studies reporting fasting plasma glucose (FPG) outcomes, the overall effects and subgroup results were not statistically significant. At 12 weeks, the pooled mean difference was −4.28 (95% CI: −8.71 to 0.16; *p* = 0.06), and at 24 weeks, it was −1.10 (95% CI: −5.05 to 2.86; *p* = 0.59). When all the studies were combined, the overall pooled mean difference was −2.61 (95% CI: −5.53 to 0.32; *p* = 0.08), indicating no significant difference in FPG levels between the experimental and control groups. Heterogeneity across the studies was substantial (I^2^ = 73%), suggesting notable variability in effect estimates. The test for subgroup differences by follow-up duration was not statistically significant (*p* = 0.29), indicating that the length of follow-up did not significantly influence the treatment effect (Figure 2).

### 3.5. Insulin Level

**Figure 3:**
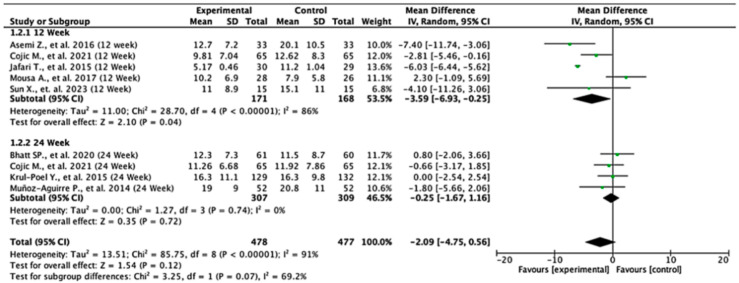
Forest plot for insulin level outcome. All studies shown are referenced as: Asemi Z., et al., (2016), Cojic M., et al., (2021), Jafari T., et al., (2015), Mousa A., et al., (2017), Sun X., et al., (2023), Bhatt SP., et al., (2020), Krul-Poel Y., et al., (2015), Muñoz-Aguirre P., et al., (2014) [26,27,28,29,30,31,32,33].

In the 12-week follow-up subgroup, the pooled mean difference was −3.59 units (95% CI: −6.93 to −0.25; *p* = 0.04), indicating a statistically significant reduction in insulin levels favoring the experimental intervention. This suggests a modest short-term benefit. However, no significant effect was observed in the 24-week subgroup, with a pooled mean difference of −0.25 units (95% CI: −1.67 to 1.16; *p* = 0.72). The overall pooled analysis across all the studies yielded a mean difference of −2.09 units (95% CI: −4.75 to 0.56; *p* = 0.12), showing no statistically significant difference between the intervention and control groups. Substantial heterogeneity was present (I^2^ = 91%), likely due to differences in study design, populations, or intervention protocols. The test for subgroup differences between the 12-week and 24-week groups was not statistically significant (*p* = 0.07), suggesting that follow-up duration alone does not explain the observed variability in treatment effect. These findings indicate potential early benefits that may not be sustained over time, highlighting the need for further high-quality studies to clarify the long-term effects of the intervention on insulin levels (Figure 3).

### 3.6. HOMA-IR

**Figure 4:**
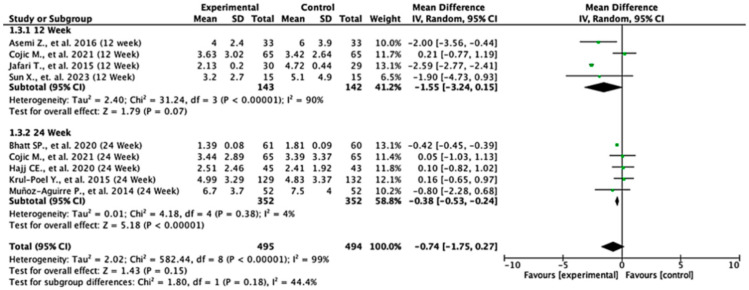
Forest plot for HOMA-IR outcome. All studies shown are referenced as: Asemi Z., et al., (2016), Cojic M., et al., (2021), Jafari T., et al., (2015), Sun X., et al., (2023), Bhatt SP., et al., (2020), Hajj CE., et al., (2020), Krul-Poel Y., et al., (2015), Muñoz-Aguirre P., et al., (2014) [20,26,27,28,29,31,32,33].

Among the studies reporting HOMA-IR outcomes, the treatment effects varied across follow-up durations. At 12 weeks, the pooled mean difference was −1.55 (95% CI: −3.24 to 0.15; *p* = 0.07), indicating no statistically significant difference between the groups. In contrast, at 24 weeks, the pooled mean difference was −0.38 (95% CI: −0.53 to −0.24; *p* < 0.00001), demonstrating a significant reduction in HOMA-IR in favor of the experimental group. When all the studies were combined, the overall pooled mean difference was −0.74 (95% CI: −1.75 to 0.27; *p* = 0.15), suggesting no statistically significant difference overall. Moderate heterogeneity was observed (I^2^ = 59%), indicating variability between study results. The test for subgroup differences between 12- and 24-week follow-ups was not statistically significant (*p* = 0.18), suggesting that follow-up duration did not significantly modify the treatment effect (Figure 4).

### 3.7. HOMA-B

**Figure 5:**
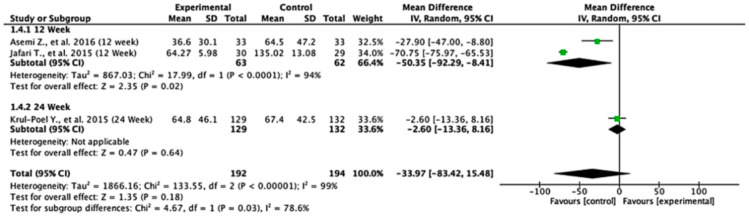
Forest plot for HOMA-B outcome. All studies shown are referenced as: Asemi Z., et al., (2016), Jafari T., et al., (2015), Krul-Poel Y., et al., (2015) [27,28,29].

Across the studies reporting HOMA-B outcomes, the effects varied between follow-up durations. At 12 weeks, the pooled mean difference was −50.35 (95% CI: −92.29 to −8.41; *p* = 0.02), indicating a statistically significant difference favoring the control group. At 24 weeks, the pooled mean difference was −2.60 (95% CI: −13.36 to 8.16; *p* = 0.64), showing no significant difference between the experimental and control groups. When all the studies were combined, the overall mean difference was −33.97 (95% CI: −83.42 to 15.48; *p* = 0.18), suggesting no statistically significant effect. Heterogeneity was extremely high (I^2^ = 99%), reflecting substantial variation across the studies. The test for subgroup differences was statistically significant (*p* = 0.03), indicating that follow-up duration significantly influenced the treatment effect (Figure 5).

### 3.8. HbA1c Level

**Figure 6:**
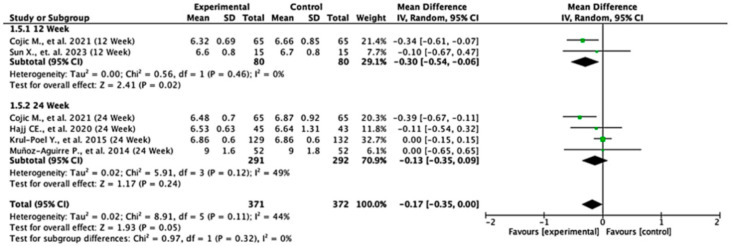
Forest plot for HbA1C outcome. All studies shown are referenced as: Cojic M., et al., (2021), Sun X., et al., (2023), Hajj CE., et al., (2020), Krul-Poel Y., et al., (2015), Muñoz-Aguirre P., et al., (2014) [20,26,28,32,33].

Among the studies reporting HbA1c outcomes, the treatment effects varied between follow-up durations. At 12 weeks, the pooled mean difference was −0.30 (95% CI: −0.54 to −0.06; *p* = 0.02), indicating a statistically significant reduction in HbA1c favoring the experimental group. At 24 weeks, the pooled mean difference was −0.13 (95% CI: −0.35 to 0.09; *p* = 0.24), showing no significant difference between the groups. When all the studies were combined, the overall pooled mean difference was −0.17 (95% CI: −0.35 to 0.00; *p* = 0.05), suggesting a borderline effect favoring the experimental treatment. Moderate heterogeneity was observed (I^2^ = 44%), indicating some variability across the studies. The test for subgroup differences by follow-up duration was not statistically significant (*p* = 0.32), suggesting that treatment effect was not dependent on follow-up time (Figure 6).

### 3.9. Hs-CRP

**Figure 7:**
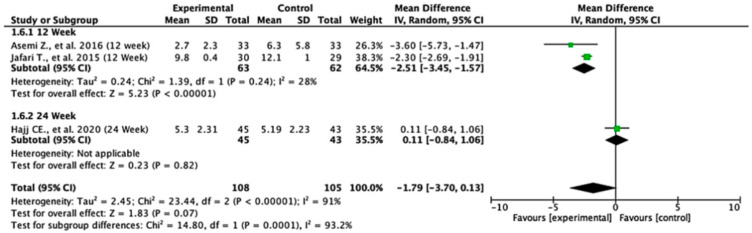
Forest plot for Hs-CRP outcome. All studies shown are referenced as: Asemi Z., et al., (2016), Jafari T., et al., (2015), Hajj CE., et al., (2020) [20,27,29].

Among the studies reporting hs-CRP outcomes, the treatment effects differed across follow-up durations. At 12 weeks, the pooled mean difference was −2.51 (95% CI: −3.45 to −1.57; *p* < 0.00001), indicating a statistically significant reduction in hs-CRP levels favoring the experimental group. At 24 weeks, the pooled mean difference was 0.11 (95% CI: −0.84 to 1.06; *p* = 0.82), showing no significant difference between the two groups. The overall pooled mean difference across all the studies was −1.79 (95% CI: −3.70 to 0.13; *p* = 0.07), suggesting a trend toward significance in favor of the experimental treatment. Heterogeneity was high (I^2^ = 91%), indicating considerable variability among studies. The test for subgroup differences was statistically significant (*p* = 0.0001), suggesting that the follow-up duration significantly influenced the treatment effects (Figure 7).

### 3.10. Level Change in Serum Vitamin D from Baseline

**Figure 8:**
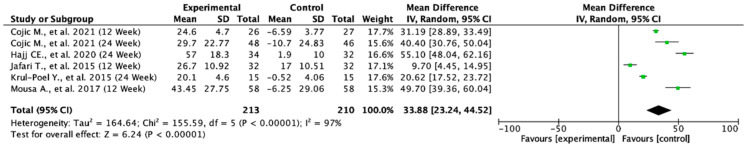
Forest plot for the mean difference from baseline of serum vitamin D. All studies shown are referenced as: Cojic M., et al., (2021), Hajj CE., et al., (2020), Jafari T., et al., (2015), Krul-Poel Y., et al., (2015), Mousa A., et al., (2017) [20,27,28,30,32].

Across the included studies, the pooled mean difference in serum vitamin D levels was 33.88 (95% CI: 23.24 to 44.52; *p* < 0.00001), indicating a statistically significant increase in favor of the experimental group. This finding suggests that the intervention substantially improved serum vitamin D levels compared to the controls. Heterogeneity was very high (I^2^ = 97%), reflecting considerable variability across the studies, which may be attributed to differences in population characteristics, baseline vitamin D status, dosage, or treatment duration. Despite the high heterogeneity, the consistent direction of effect across the studies supports the robustness of the observed benefit (Figure 8).

### 3.11. GRADE Analysis

The quality of evidence for each outcome was systematically evaluated using the GRADE approach, considering five domains: risk of bias, inconsistency, indirectness, imprecision, and publication bias. The results are summarized below.

For fasting plasma glucose (FPG), the certainty of evidence was rated as low. Although a trend toward reduced glucose levels was observed (MD: −2.61 mg/dL), the confidence interval crossed the null, and substantial heterogeneity was detected (I^2^ = 73%). Additionally, three out of nine studies exhibited unclear randomization procedures, contributing to a moderate risk of bias and subsequent downgrading for both inconsistency and imprecision (Table 3).

The outcome for insulin level also received a low certainty rating, with no statistically significant effect (MD: −2.09; *p* = 0.12) and considerable heterogeneity (I^2^ = 91%). The presence of unclear randomization in several studies further raised concerns about internal validity. Consequently, the evidence was downgraded for both inconsistency and imprecision.

For HOMA-IR, the quality of evidence was likewise rated as low. The pooled effect (MD: −0.74; *p* = 0.15) was not statistically significant, and the confidence interval included the null. Although heterogeneity was moderate (I^2^ = 59%), it still warranted downgrading for inconsistency in addition to imprecision. Risk of bias concerns persisted due to unclear allocation procedures in a subset of studies.

The certainty of evidence for HOMA-B was deemed very low, reflecting substantial uncertainty. The effect size was highly variable (MD: −33.97), with a wide confidence interval and extreme heterogeneity (I^2^ = 99%). Additionally, a significant subgroup difference (*p* = 0.03) suggests possible effect modification by follow-up duration. These issues, along with a moderate risk of bias, led to downgrading across three domains: inconsistency, imprecision, and subgroup inconsistency.

In contrast, HbA1c level was supported by moderate-certainty evidence, with a borderline significant reduction (MD: −0.17; *p* = 0.05) and acceptable heterogeneity (I^2^ = 44%). The evidence was downgraded only for imprecision, as the confidence interval approached the null, but the risk of bias was generally low across most studies.

The analysis for Hs-CRP showed a potential trend toward improvement (MD: −1.79; *p* = 0.07) but was supported by low-certainty evidence due to high heterogeneity (I^2^ = 91%), imprecision, and a significant subgroup effect (*p* = 0.0001). Additionally, randomization concerns in some studies contributed to the overall moderate risk of bias.

Finally, serum vitamin D change from baseline was the only outcome with a clearly favorable and statistically significant effect (MD: +33.88; *p* < 0.00001). Despite very high heterogeneity (I^2^ = 97%), the consistency in effect direction and magnitude across the studies justified a moderate certainty rating. Most included trials were assessed as having a low to moderate risk of bias, and the large effect size strengthened confidence in the result despite some variability.

### 3.12. Leave-One-Out Sensitivity Analysis

The leave-one-out sensitivity analysis helped us better understand how individual studies influenced the overall findings for fasting plasma glucose (FPG), insulin level, HOMA-IR, HOMA-B, HbA1c, hs-CRP, and serum vitamin D level change outcomes.

**Figure 9:**
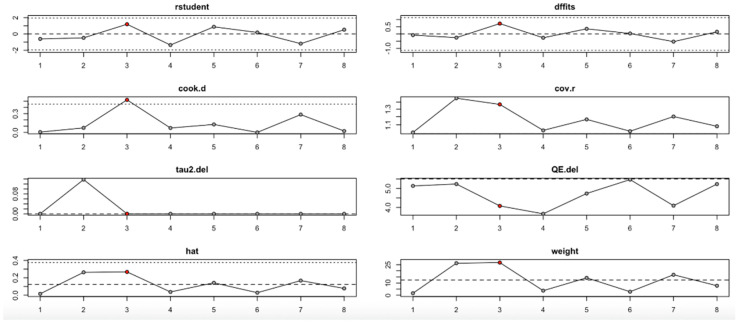
Baujat plot for fasting plasma glucose outcome.

For FPG (Figure 9, Table 4), heterogeneity was moderate to high, with I^2^ values fluctuating between approximately 42.7% and 90.9% across iterations. Notably, omitting certain studies led to reductions in heterogeneity, but the pooled effect estimates remained relatively consistent. The third study exerted a notable influence, as indicated by elevated values in studentized residuals, Cook’s distance, DFFITS, and hat diagnostics, suggesting some sensitivity to that study. However, the overall statistical conclusions were robust.

**Figure 10:**
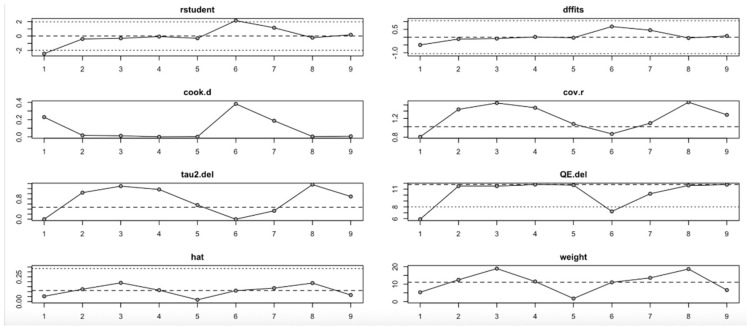
Baujat plot for insulin level outcome.

For the insulin level outcome (Figure 10, Table 4), the I^2^ values remained substantial across all iterations, indicating persistent heterogeneity even after sequential study omission. Although no single study drastically altered the pooled effect estimate, moderate shifts in tau^2^ and Q statistics were observed, particularly when omitting studies with higher Cook’s distance and hat values. This implies that while individual studies exert moderate influence, the insulin level findings are relatively stable.

**Figure 11:**
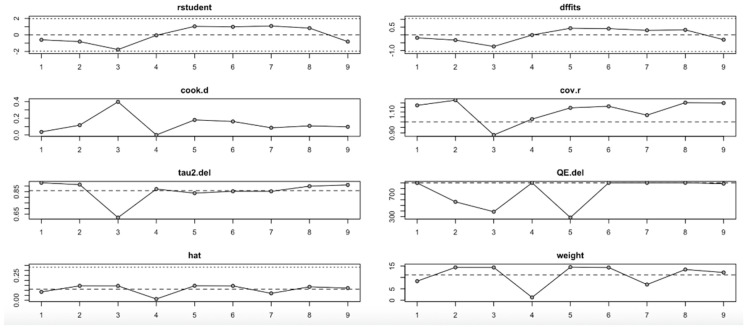
Baujat plot for HOMA-IR outcome.

In the HOMA-IR outcome (Figure 11, Table 4), the I^2^ values ranged from low to moderate (20–77%), with only slight changes across iterations. No single study had a disproportionate impact on the effect size or heterogeneity. Influence diagnostics (e.g., Cook’s distance, DFFITS, hat) remained within acceptable limits, suggesting that the pooled findings for HOMA-IR are robust and not strongly driven by any outlier study.

**Figure 12:**
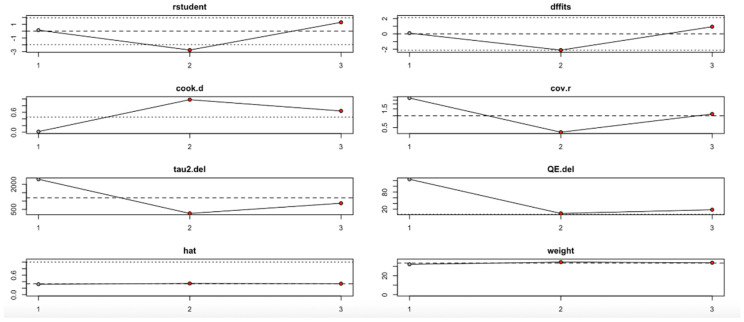
Baujat plot for HOMA-B outcome.

Conversely, the HOMA-B outcome (Figure 12, Table 4) showed high sensitivity to the second study. Omitting this study significantly affected the effect estimate and dramatically reduced heterogeneity (I^2^), tau^2^, and Q values. Influence diagnostics such as Cook’s distance and DFFITS confirmed this study’s substantial influence. Despite these variations, the overall effect direction remained consistent, although the robustness of the findings is limited by the small number of included studies.

**Figure 13:**
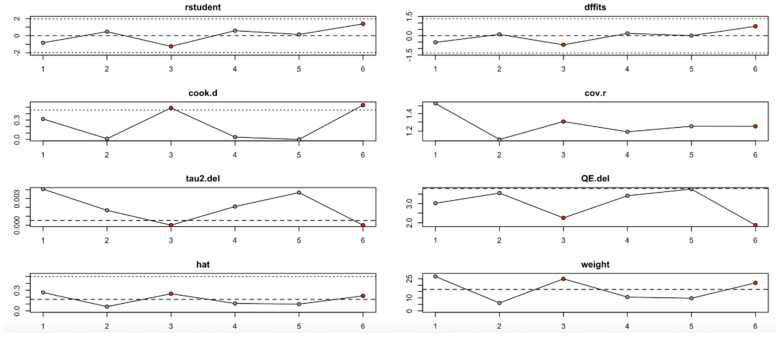
Baujat plot for HbA1c level outcome.

For the HbA1c level outcome (Figure 13, Table 4), heterogeneity remained moderate throughout the leave-one-out iterations. The sixth study appeared particularly influential, as its removal altered both the pooled estimate and the heterogeneity statistics (e.g., reduction in QE.del and tau^2^). Diagnostic values supported its influence, but even with its exclusion, the effect remained statistically robust.

**Figure 14:**
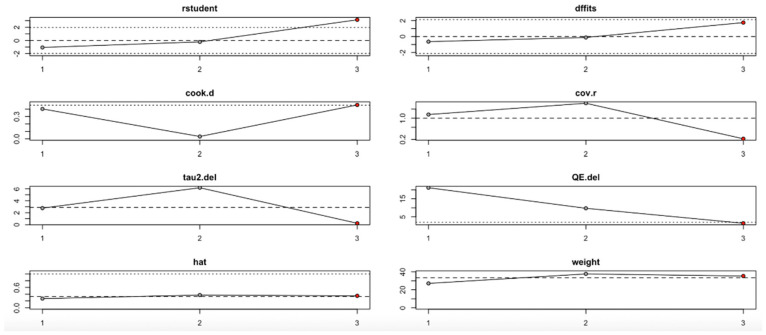
Baujat plot for hs-CRP level outcome.

The hs-CRP outcome (Figure 14, Table 4) revealed that the third study had an outsized impact. Its exclusion resulted in large reductions in heterogeneity (I^2^) and tau^2^, and influence diagnostics exceeded typical thresholds. This suggests that the findings for hs-CRP may not be fully stable and should be interpreted cautiously.

**Figure 15:**
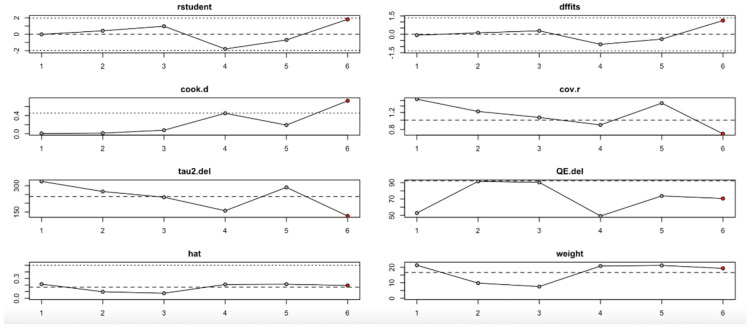
Baujat plot for level change in serum Vitamin D from Baseline outcome.

Finally, the serum vitamin D level change (Figure 15, Table 4) showed one particularly influential study (study 6), which notably shifted the tau^2^ and heterogeneity metrics. Diagnostic plots confirmed its high leverage and influence. Nevertheless, the pooled estimate remained statistically significant, indicating that the beneficial effect on vitamin D level is relatively robust, albeit with some sensitivity to this outlier.

In summary, the leave-one-out analysis indicates that while most outcomes (FPG, insulin, HOMA-IR, HbA1c, and vitamin D) show generally robust results, some outcomes—particularly HOMA-B and hs-CRP—are more sensitive to specific influential studies. This reinforces the importance of examining study-level characteristics and potential sources of heterogeneity in future research (Figure 9, Figure 10, Figure 11, Figure 12, Figure 13, Figure 14 and Figure 15, Table 4).

## 4. Discussion

This systematic review and meta-analysis assessed the impact of vitamin D supplementation on glycemic control and inflammation in patients with T2DM across nine studies. The main findings showed that vitamin D significantly reduced insulin levels, HbA1c, hs-CRP, and HOMA-B at the 12-week follow-up compared to the controls. Only HOMA-IR is observed to be statistically significant and no significant effect is found for FPG at either time point.

Starting with FPG, the studies collectively reveal a pattern where no significant differences were observed between the experimental and control groups across most follow-up periods. This result indicates that the interventions assessed may not significantly affect fasting glucose levels, at least in the timeframe examined. While the current study did not reveal a meaningful effect, earlier research reported different outcomes. A meta-analysis by Chen W et al. showed that vitamin D supplementation can significantly reduce fasting plasma glucose and the result was marked when it was given in a short-term and high dosage to patients with a vitamin D deficiency, who are overweight, or who have an HbA1C of 8% or a higher baseline [19]. The high heterogeneity observed (I^2^ = 73%) underscores the potential influence of varying study designs and population characteristics; if participants were already vitamin D sufficient, supplementation may not have yielded significant changes. A study by Pittas AG et al., showed that among populations at high risk of T2DM that are not vitamin D insufficient, vitamin D supplementation at a dose of 4000IU did not significantly lower the risk of diabetes [34]. Even with the non-significant results in this study, the potential mechanism remain relevant for future research, as vitamin D influences insulin secretion from pancreatic beta cells and potentially enhances insulin sensitivity in peripheral tissue by reducing systemic inflammation through the vitamin D receptor on pancreatic beta cells, muscles, and liver [35].

Insulin levels present a slightly different narrative. While the pooled data for the 12-week follow-up indicates a significant reduction in insulin levels favoring the experimental group, this effect does not persist at the 24-week mark. The overall trend, though leaning towards a reduction, fails to reach statistical significance, suggesting a potential short-term benefit of vitamin D on insulin levels and that the initial benefits might diminish over time. This finding aligns with the previous meta-analysis by Chen W et al., which showed that vitamin D supplementation can significantly reduce insulin levels and the result was marked when it was given in a short-term and high dosage to patients with a vitamin D deficiency, who are overweight, or who have an HbA1C of 8% or a higher level [19]. The high heterogeneity (I^2^ = 91%) again highlights the complexity of comparing studies with differing methodologies and populations.

In examining HOMA-IR, a key measure of insulin resistance, the findings reveal a mixed outcome. While no significant difference is observed at 12 weeks, the 24-week follow-up shows a meaningful reduction in insulin resistance favoring the experimental treatment. A meta-analysis by Pramono et al. showed that there was no effect of vitamin D supplementation on peripheral insulin sensitivity [36]. A different finding was found by a study by Lemieux, which showed that a 6-month vitamin D supplementation in individuals at high risk of diabetes or with newly diagnosed T2DM significantly increased peripheral insulin sensitivity [37]. This suggests that longer-term intervention may be required to observe significant improvements in insulin sensitivity.

HOMA-B, which assesses beta cell function, tells yet another story. The 12-week follow-up data suggest that the control group may experience better outcomes, as indicated by a significant difference favoring the control. However, by 24 weeks, these differences are no longer significant, indicating that any early advantage might not persist or could even reverse over time. The high heterogeneity (I^2^ = 99%) here is particularly striking, reflecting substantial variability across the studies.

The HbA1c levels, a long-term marker of blood glucose control, show a small but significant reduction at 12 weeks in favor of the experimental group. However, this difference does not remain significant at 24 weeks. Our finding does not align with a study by Cojic M et al., which reported that oral daily vitamin D supplementation significantly improved HbA1c levels over a 3-month and 6-month period [32]. However, our finding aligns with a meta-analysis by Hu et al., which reported that vitamin D supplementation in T2D patients significantly improved Hba1c in a short-term intervention (<6 months) [38].

Analysis of hs-CRP reveals that the experimental treatments are associated with significant reductions at 12 weeks, though these effects do not persist at 24 weeks. The overall trend suggests that the treatments may have an anti-inflammatory effect, but this is not sustained over time. A meta-analysis by Yu et al. showed that vitamin D supplementation over 12 weeks is significantly beneficial for the reduction of hs-CRP in T2DM patients; however, this study also has high heterogeneity [39]. The high heterogeneity (I^2^ = 91%) in our study again suggests variability in study designs and populations, which complicates the interpretation of these findings.

The analysis of serum vitamin D level changes from baseline demonstrates a significant increase in vitamin D levels favoring the experimental group, indicating that supplementation effectively improves vitamin D status. This result is consistent with previous studies showing that vitamin D supplementation reliably raises serum levels, particularly when higher doses are used over shorter periods. However, the very high heterogeneity (I^2^ = 97%) highlights substantial differences between the studies, likely due to variations in baseline vitamin D levels, dosage, treatment duration, and population characteristics. This variability reflects the well-documented observation that individuals with lower baseline vitamin D levels, higher BMI, or metabolic disturbances tend to show a more pronounced response to supplementation. While the biochemical efficacy of vitamin D supplementation is clear, its clinical relevance in relation to glycemic control and inflammatory outcomes remains uncertain. This finding reinforces the need for future research to focus not only on correcting deficiency but also on determining whether these improvements lead to meaningful metabolic benefits, especially in populations at higher risk of type 2 diabetes. Future trials should aim for longer follow-up periods, standardized dosing protocols, and better participant stratification based on baseline vitamin D status and metabolic risk to better understand the long-term effects of supplementation.

This study’s strengths include its comprehensive approach as the first systematic review and meta-analysis focusing on the impact of vitamin D3 supplementation on key biomarkers related to T2DM, such as FPG, insulin levels, HOMA-IR, HOMA-B, HbA 1c, and hs-CRP. It also benefits from including data from diverse global studies, enhancing the generalizability of the findings. Given these outcomes, clinicians might consider short- to medium-term vitamin D supplementation for T2DM patients who are vitamin D deficient, especially to target inflammatory markers and short-term glucose improvements. However, the diminishing effects over time warrant cautious interpretation. From a research perspective, the findings highlight the need for longer-duration trials and exploration into the dose–response relationship to determine the optimal strategy for sustained metabolic benefits.

This study has several limitations. Firstly, many of these studies employed short follow-up periods, which may limit the ability to evaluate the long-term effects or sustainability of the benefit of vitamin D. Also, variability in population characteristics, such as age, BMI, and baseline vitamin D levels, further influences the outcomes and may limit applicability to specific subgroups. The geographic and healthcare availability among the study populations (ranging from Iran to Mexico to Australia) may also contribute to heterogeneity in outcomes due to differences in diabetes management standards, diet, sun exposure, and healthcare access. The baseline vitamin D status was also not consistently stratified across the studies, which could affect how the participants respond to supplementation and make it harder to see differences between specific groups.

Future research should prioritize long-term randomized controlled trials (exceeding six months), stratification of study populations based on baseline vitamin D status, HbA1c, and BMI, and the standardization of dosing regimens. Further investigations should also examine the underlying mechanisms of action, such as immune modulation, and explore the synergistic effects of vitamin D when combined with other antidiabetic treatments. These directions will help identify the subgroups most likely to benefit and inform more personalized, evidence-based strategies for vitamin D use in T2DM management.

## 5. Conclusions

The analysis of various biomarkers and the evidence from multiple studies suggests that while vitamin D supplementation shows potential benefits in managing T2DM, these effects are complex and may require individualized approaches. The observed trends in FPG, insulin levels, HOMA-IR, HOMA-B, HbA1c, and hs-CRP indicate that vitamin D may influence glycemic control, insulin sensitivity, and inflammation, but these effects are often modest and may diminish over time. The variability in outcomes across the studies highlights the importance of considering factors such as baseline vitamin D levels, dosage, duration of treatment, and individual patient characteristics. High-dose vitamin D regimens might be necessary for rapid correction in certain populations, while maintenance doses could sustain benefits in the longer term. However, the heterogeneity observed in the data suggests that further research is needed to fully understand the optimal long-term implications of vitamin D supplementation in T2DM management.

## Figures and Tables

**Table 1:** Database search keywords.

Database	Combined Keywords with Boolean Operators	Filter Applies
PubMed	(“Vitamin D” OR Calcitriol) AND (adult OR obesity) AND “Diabetes Mellitus Type 2”	- Publication date: Last 10 years - Article type: Randomized Controlled Trial (RCT) and Clinical Trial (CT)
Scopus	TITLE-ABS-KEY (“Vitamin D” OR Calcitriol) AND (adult OR obesity) AND “Diabetes Mellitus Type 2”	- Publication date: Last 10 years - Document type: Article (Research Article)
Cochrane Library	(“Vitamin D” OR Calcitriol) AND (adult OR obesity) AND “Diabetes Mellitus Type 2”: ti, ab, kw	- Publication date: Last 10 years - Study type: Randomized Controlled Trial (RCT) and Clinical Trial (CT)
Epistemonikos	(“Vitamin D” OR Calcitriol) AND (adult OR obesity) AND “Diabetes Mellitus Type 2”	- Publication date: Last 10 years - Study type: Randomized Controlled Trial (RCT) and Clinical Trial (CT)
ScienceDirect	TITLE-ABSTR-KEY (“Vitamin D” OR Calcitriol) AND (adult OR obesity) AND “Diabetes Mellitus Type 2”)	- Publication date: Last 10 years - Document type: Research Article

**Table 2:** Study characteristics.

Author; Year	Country/ Place	Study Design	Vitamin D Level Before Treatment (Intervention/Control)	Vitamin D Level Before Treatment (Intervention/Control)	Supplementation	Population				Functional Outcome (Intervention/Control) (Mean ± SD)						
						Sample Size (*n*)	Supplementation/Placebo	Mean Age (Years) (Mean ± SD)	Follow-Up (Weeks)	FPG	Insulin	HOMA-IR	HOMA-B	HbA1c	Hs-CRP	TNF-a
Muñoz, et al.; 2014 [26]	Mexico	Randomized Clinical Trial	Not Reported	Not Reported	vit D3 4000 IU/day	104	52/52	Supplementation: 56.1 ± 5.1; Placebo: 57.4 ± 5.0	24 weeks	146 ± 40/154 ± 42	19.0 ± 9.0/20.8 ± 11.0	6.7 ± 3.7/7.5 ± 4.0	No Result	9.0 ± 1.6/9.0 ± 1.8	No Result	No Result
Jafari., et al.; 2015 [27]	Iran	Randomized Clinical Trial	62.23 ± 4.52/62.72 ± 4.27	86.83 ± 4.87/56.13 ± 2.89	2000 IU vitamin D3 in 100 g/day	59	30/29	Supplementation: 57.8 ± 5.5; Placebo: 56.8 ± 5.7	12	No Result	5.17 ± 0.46/11.20 ± 1.04	2.13 ± 0.20/4.72 ± 0.44	64.27 ± 5.98/135.02 ± 13.08	7.24 ± 0.22/7.58 ± 0.23	9.8 ± 0.4/12.1 ± 1.0	No Result
Krul., et al.; 2015 [28]	Netherlands	Randomized Clinical Trial	60.6 ± 23.3/59.1 ± 23.2	90.3 ± 22.2/48.4 ± 26.2	vit D3 50,000 IU/month	261	129/132	Supplementation: 67 ± 8; Placebo: 67 ± 9	24	8.1 ± 1.4/7.8 ± 1.2	16.3 (11.1)/16.3 (9.8)	4.99 (3.29–6.98)/4.83 (3.37–7.24)	64.8 (46.1–93.8)/67.4 (42.5–102.7)	6.86 (0.6)/6.86 (0.6)	No Result	No Result
Asemi., et al.; 2016 [29]	Iran	Randomized Clinical Trial	Not Reported	Not Reported	5 μg vit D3, 90 μg vit K plus 500 mg Ca	66	33/33	Supplementation: 65.9 (11.4); Placebo: 65.0 (11.1)	12	140.7 (56.2)/145·5 (70.4)	12.7 (7.2)/20.1 (10.5)	4.0 (2.4)/6.0 (3.9)	36.6 (30.1)/64.5 (47.2)	No Result	2.7 (2.3)/6.3 (5.8)	No Result
Mousa., et al.; 2017 [30]	Australia	Randomized Clinical Trial	31.4 ± 12.6/34.2 ± 10.0	88.4 ± 21.0/36.1 ± 10.0	vit D3 100,000 IU bolus followed by 4000 IU/day	54	28/26	Supplementation: 30.5 (25–35); Placebo: 29.5 (25–41)	16	4.6 ± 0.5/4.7 ± 0.3	10.2 (6.9–18.2)/7.9 (5.8–13.6)	No Result	No Result	No Result	No Result	No Result
Bhatt., et al.; 2020 [31]	India	Randomized Clinical Trial	29.9 ± 5.9/32.1 ± 5.2	56.6 ± 12.6/49.1 ± 12.1	vit D3 60,000 IU/week	121	61/60	Not stated	26	110.9 ± 8.5/111.8 ± 7.9	12.3 ± 7.3/11.5 ± 8.7	1.39 ± 0.08/1.81 ± 0.09	No Result	No Result	No Result	No Result
									52	108.4 ± 21.0/105.5 ± 17.5	10.7 ± 5.6/10.3 ± 4.8	1.42 ± 0.09/1.38 ± 0.07	No Result	No Result	No Result	No Result
									78	101.8 ± 11.9/106.9 ± 26.9	10.6 ± 4.8/10.1 ± 4.7	1.20 ± 0.06/1.24 ± 0.04	No Result	No Result	No Result	No Result
Hajj., et al.; 2020 [20]	Lebanon	Randomized Clinical Trial	14.8 ± 4.5/15.02 ± 4.2	34.9 ± 4.7/14.5 ± 3.9	vit D3 30,000 IU/week	88	45/43	Supplementation: 66.9 ± 4.1; Placebo: 65.7 ± 4.5	24	No Result	No Result	2.51 ± 2.46/2.41± 1.92	No Result	6.53 ± 0.63/6.64 ± 1.31	5.3 ± 2.31/5.19 ± 2.23	2.61 ± 1.04/3.01 ± 0.83
Cojic., et al.; 2021 [32]	Serbia	Randomized Clinical Trial	48.79 ± 31.63/58.02 ± 32.32	92.24 ± 20.25/51.77 ± 23.99	vit D3 14,000 IU/week	130	65/65	Supplementation: 60.41 (8.5); Placebo: 63.65 (8.2)	12	7.28 (1.16)/7.97 (1.74)	9.81 (7.04)/12.62 (8.30)	3.63 (3.02)/3.42 (2.64)	No Result	6.32 (0.69)/6.66 (0.85)	No Result	No Result
									24	7.23 (1.26)/7.74 (1.49)	11.26 (6.68)/11.92 (7.86)	3.44 (2.89)/3.39 (3.37)	No Result	6.48 (0.70)/6.87 (0.92)	No Result	No Result
Sun., et al.; 2023 [33]	China	Randomized Clinical Trial	Not Reported	Not Reported	Vit D3 1000 IU/day	30	15/15	Not stated	12	6.5 ± 0.8/7.1 ± 1.3	11.0 ± 8.9/15.1 ± 11.0	3.2 ± 2.7/5.1 ± 4.9	No Result	6.6 ± 0.8/6.7 ± 0.8	No Result	No Result

**Table 3:** GRADE analysis summary result.

Outcome	Effect Size (MD, 95% CI)	No. of Studies	Risk of Bias	Certainty	Key Justification(s)
Fasting Plasma Glucose (FPG)	−2.61 [−5.53 to 0.32]; *p* = 0.08	Multiple	Moderate	⬤⬤◯◯ Low	Downgraded for inconsistency (I^2^ = 73%) and imprecision (non-significant, CI crosses 0); 3/9 studies had unclear randomization.
Insulin Level	−2.09 [−4.75 to 0.56]; *p* = 0.12	Multiple	Moderate	⬤⬤◯◯ Low	Downgraded for inconsistency (I^2^ = 91%) and imprecision; risk of bias due to unclear randomization in several studies.
HOMA-IR	−0.74 [−1.75 to 0.27]; *p* = 0.15	Multiple	Moderate	⬤⬤◯◯ Low	Downgraded for imprecision (CI crosses 0) and moderate inconsistency (I^2^ = 59%); some studies with unclear randomization.
HOMA-B	−33.97 [−83.42 to 15.48]; *p* = 0.18	Multiple	Moderate	⬤◯◯◯ Very Low	Downgraded for inconsistency (I^2^ = 99%), imprecision (wide CI), and subgroup inconsistency (*p* = 0.03); moderate RoB due to unclear randomization.
HbA1c Level	−0.17 [−0.35 to 0.00]; *p* = 0.05	Multiple	Low–Moderate	⬤⬤⬤◯ Moderate	Downgraded for imprecision (borderline *p* and CI), but heterogeneity was acceptable (I^2^ = 44%); most studies low risk, some concerns in 3/9 studies.
Hs-CRP	−1.79 [−3.70 to 0.13]; *p* = 0.07	Multiple	Moderate	⬤⬤◯◯ Low	Downgraded for inconsistency (I^2^ = 91%), imprecision, and significant subgroup inconsistency (*p* = 0.0001); 3 studies had randomization issues.
Serum Vitamin D Change	+33.88 [23.24 to 44.52]; *p* < 0.00001	Multiple	Low–Moderate	⬤⬤⬤◯ Moderate	Downgraded for inconsistency (I^2^ = 97%) but retained moderate certainty due to consistent large effect and direction despite some RoB concerns.

**Table 4:** Leave-one-out sensitivity analysis summary.

Outcome	Leave-One-Out	Estimate	SE	Z-Value	*p*-Value	CI Lower	CI Upper	Q	Qp	Tau^2^	I^2^ (%)	H^2^
FPG	−1	6.867	2.068	−1.979	0.304	2.359	5.212	5.638	0.912	8.413	90.926	5.629
	−2	7.895	1.935	4.247	0.05	−3.033	9.945	18.72	0.317	6.322	88.636	7.969
	−3	9.817	2.595	0.679	0.005	2.17	14.966	1.162	0.2	5.852	20.305	4.857
	−4	4.199	1.267	−2.145	0.231	−0.436	11.977	7.795	0.717	9.27	46.479	4.551
	−5	7.814	1.412	−1.684	0.071	−1.755	6.209	1.35	0.231	8.277	76.697	6.229
	−6	4.584	0.978	2.348	0.763	4.319	10.332	17.03	0.376	5.789	75.2	8.259
	−7	8.265	2.725	0.398	0.565	−2.31	6.406	12.79	0.511	7.468	45.921	3.479
	−8	−0.421	2.354	−0.238	0.025	−3.871	11.242	2.871	0.138	3.745	63.509	9.057
Insulin	−1	9.43	1.695	1.836	0.589	2.31	9.904	8.948	0.21	1.728	74.026	5.277
	−2	8.679	2.101	−1.812	0.285	4.101	10.692	10.87	0.35	9.945	88.868	6.705
	−3	8.833	2.238	−0.409	0.135	2.425	5.672	8.345	0.637	2.751	74.423	7.825
	−4	7.029	2.028	−0.259	0.271	1.782	5.36	16.77	0.913	8.861	10.761	7.827
	−5	−1.348	1.947	4.826	0.864	1.021	12.628	0.842	0.433	2.936	76.555	3.903
	−6	6.251	2.966	0.188	0.882	−2.605	9.518	19.01	0.031	8.053	74.418	1.281
	−7	4.498	0.867	4.576	0.632	2.771	5.619	12.94	0.451	3.922	30.152	8.383
	−8	0.405	2.904	2.965	0.745	1.097	5.185	6.329	0.273	7.27	29.534	8.056
	−9	3.272	0.627	−2.857	0.374	−1.144	6.785	19.51	0.456	8.888	20.875	8.239
HOMA-IR	−1	−1.036	1.91	−1.502	0.009	2.614	11.05	0.769	0.553	1.371	96.687	3.014
	−2	6.221	2.305	1.37	0.185	−2.339	10.231	6.836	0.599	4.722	11.999	3.602
	−3	8.707	2.124	−1.577	0.047	0.849	8.338	15.18	0.937	6.653	17.846	7.337
	−4	5.04	1.37	2.066	0.352	−4.814	9.897	1.309	0.015	6.615	45.765	4.763
	−5	0.164	2.633	1.504	0.275	−4.673	12.593	11.6	0.407	0.734	76.639	6.544
	−6	−1.884	0.971	1.925	0.849	4.62	12.151	4.656	0.667	8.549	20.304	4.699
	−7	2.995	0.578	−0.001	0.155	4.412	5.049	14.4	0.603	0.076	9.449	1.762
	−8	8.784	1.85	−2.758	0.049	2.173	5.831	10.33	0.042	6.437	15.698	9.872
	−9	7.155	1.255	3.718	0.387	−4.307	7.97	5.383	0.043	9.656	39.821	8.742
HOMA-B	−1	7.731	0.678	−0.314	0.788	−1.317	10.31	8.014	0.162	2.391	53.44	3.075
	−2	4.054	2.768	−1.709	0.361	−0.459	10.042	10.09	0.525	5.2	89.3	5.257
	−3	9.409	0.964	−1.253	0.53	−4.039	7.094	19.98	0.34	9.803	26.724	5.961
HbA1c	−1	0.973	1.136	4.334	0.611	3.479	8.843	14.33	0.412	3.406	38.956	1.582
	−2	2.527	2.295	0.158	0.68	2.541	12.752	9.894	0.999	1.562	99.006	5.555
	−3	5.142	0.65	−0.44	0.876	−4.328	10.953	5.21	0.494	7.379	88.052	3.918
	−4	−1.688	2.689	−0.027	0.341	2.776	12.274	17.9	0.605	1.15	73.74	6.813
	−5	5.952	1.659	3.82	0.429	3.718	11.247	17.73	0.493	9.091	55.075	3.081
	−6	1.218	1.254	−2.535	0.507	1.473	9.716	0.513	0.714	5.382	23.323	1.856
hs-CRP	−1	2.349	0.826	−1.345	0.218	−3.81	10.12	8.679	0.579	9.793	82.216	4.494
	−2	4.179	2.733	1.915	0.447	−1.445	13.693	6.918	0.859	5.061	17.348	3.277
	−3	−1.612	0.759	0.603	0.132	−1.792	13.262	13.88	0.818	6.027	29.9	4.152
Vitamin D	−1	4.048	0.724	−0.985	0.604	2.84	13.706	14.1	0.816	4.49	62.115	6.158
	−2	8.781	2.666	−1.18	0.208	0.56	8.886	4.445	0.69	6.638	79.21	5.533
	−3	3.285	2.569	−1.709	0.303	1.48	9.355	14.12	0.085	3.186	87.486	1.038
	−4	3.479	1.634	2.203	0.197	−2.116	9.983	11.58	0.868	9.388	71.056	8.379
	−5	−0.021	2.059	−2.096	0.05	4.012	5.333	11.82	0.253	4.188	5.06	4.221
	−6	0.712	2.991	−1.793	0.591	−3.136	9.245	19.24	0.299	1.389	22.088	6.045

## Data Availability

The original data presented in the study are openly available in Supplementary File S1.

## Supplementary Materials

The following supporting information can be downloaded at: https://www.mdpi.com/article/10.3390/nu17152489/s1, Supplementary File S1_PRISMA Checklist; Supplementary File S2_Studies Screening Report; Supplementary File S3_Risk of Bias Assessment.
