# Supplementary material for: The Impact of Vitamin D Supplementation on Fasting Plasma Glucose, Insulin Sensitivity, and Inflammation in Type 2 Diabetes Mellitus: A Systematic Review and Meta-Analysis
Source: Nutrients. 2025 Jul 30;17(15):2489. doi: 10.3390/nu17152489 (PMC12348612; doi:10.3390/nu17152489)
Supplement: Supplementary file 1 [file nutrients-17-02489-s001.zip › Supplementary File S2_Studies Screening Report.pdf]

N0	Authors	Title	Included in the review	Reason of exclusion
1.	Muñoz-Aguirre et al., 2015	The effect of vitamin D supplementation on serum lipids in postmenopausal women with diabetes: A randomized controlled trial	Yes	-
2.	Jafari et al., 2015	Effects of vitamin D-fortified low fat yogurt on glycemic status, anthropometric indexes, inflammation, and bone turnover in diabetic postmenopausal women: A randomised controlled clinical trial	Yes	-
3.	Krul-Poel et al., 2015	Effect of Vitamin D Supplementation on Glycemic Control in Patients With Type 2 Diabetes (SUNNY Trial)	Yes	-
4.	Asemi et al., 2016	The effects of vitamin D, K and calcium co-supplementation on carotid intima-media thickness and metabolic status in overweight type 2 diabetic patients with CHD	Yes	-
5.	Mousa et al., 2017	25-hydroxyvitamin D is associated with adiposity and cardiometabolic risk factors in a predominantly vitamin D-deficient and overweight/obese but otherwise healthy cohort	Yes	-
6.	Bhatt et al., 2020	Vitamin D Supplementation in Overweight/obese Asian Indian Women with Prediabetes	Yes	-
7.	Hajj et al., 2020	Effect of Vitamin D Supplementation on Inflammatory Markers in Non-Obese Lebanese Patients with Type 2 Diabetes: A Randomized Controlled Trial	Yes	-
8.	Sun et al., 2023	Effects of Endurance Exercise and Vitamin D Supplementation on Insulin Resistance and Plasma Lipidome in Middle-Aged Adults with Type 2 Diabetes	Yes	-
9.	Cojic et al., 2021	The Effects of Vitamin D Supplementation on Metabolic and Oxidative Stress Markers in Patients With Type 2 Diabetes: A 6-Month Follow Up Randomized Controlled Study	Yes	-
10.	Zhao et al., 2024	The association between vitamin D receptor gene polymorphism FokI and type 2 diabetic kidney disease	No	irrelevant

11.	Qian et al., 2024	Biomarkers of glucose-insulin homeostasis and incident type 2 diabetes and cardiovascular disease	No	different outcomes
12.	Hassan et al., 2024	Bone Health in Patients with Rheumatoid Arthritis in Bahrain	No	irrelevant
13.	Dhaher et al., 2024	Impact of lifestyle intervention on vitamin D, Adiponectin, IGF-1 and Proneurotensin in overweight	No	different outcomes
14.	Mitu et al., 2024	Analysis of genetic association of vitamin D receptor (VDR) gene FokI polymorphism in T2DM patients	No	irrelevant
15.	Haramshahi et al., 2024	Nutrient patterns and risk of diabetes mellitus type 2: a case-control study	No	different outcomes
16.	Wang et al., 2024	Heterogeneous enhancer states orchestrate β cell responses to metabolic stress	No	irrelevant
17.	Ma et al., 2024	Vitamin D level as a predictor of dysmobility syndrome with type 2 diabetes	No	different outcomes
18.	Parveen et al., 2024	Delving the vitamin D receptor variation and expression profiles in the context of T2DM among families	No	irrelevant
19.	Fang et al., 2024	Relationship between NAFLD and serum 25-hydroxy vitamin D in T2DM patients	No	different outcomes
20.	Gholami et al., 2024	The effect of BsmI SNP of VDR on insulin resistance in healthy children and adolescents	No	different outcomes
21.	Chen et al., 2024	Efficacy of vitamin D supplementation on glycaemic control in type 2 diabetes	No	irrelevant
22.	Ahsan et al., 2024	Vitamin D status of patients visiting a private endocrinology clinic in Pakistan	No	irrelevant
23.	Chen et al., 2024	Vitamin D deficiency and the risk of diabetic retinopathy in patients with type 2 diabetes	No	different outcomes
24.	Mondal et al., 2024	A systematic review of the major risk factors for type two diabetes among Aboriginal Australians	No	different outcomes
25.	Sarma et al., 2024	Weight change, variability, and hip fracture risk in older adults with dysglycemia	No	different outcomes

26.	Guido et al., 2024	Association between liver steatosis, fibrosis, and onset of T2DM in overweight individuals	No	different outcomes
27.	Muscogiuri et al., 2024	Impact of COVID-19 pandemic on diet and physical activity among Latinos of Mexican ancestry	No	irrelevant
28.	Zhang et al., 2024	Association of polychlorinated biphenyls with vitamin D among rural Chinese adults	No	irrelevant
29.	Xiang et al., 2024	Elevated TyG index is associated with increased risk of vitamin D deficiency in elderly with T2DM	No	different outcomes
30.	Johnson et al., 2023	The long-term effect of intentional weight loss on changes in bone mineral density in persons with type 2 diabetes: results from the Look AHEAD randomized trial	No	different outcomes
31.	He et al., 2023	A Mendelian randomization study on causal effects of 25(OH) vitamin D levels on diabetic nephropathy	No	different outcomes
32.	Fenercioglu et al., 2023	The Association between Serum 25-Hydroxyvitamin D3 Levels and Pro-Inflammatory Markers in New-Onset Type 2 Diabetes Mellitus and Prediabetes	No	irrelevant
33.	Ghosh et al., 2023	Sedentary lifestyle with increased risk of obesity in urban adult academic professionals: an epidemiological study in West Bengal, India	No	irrelevant
34.	Wee et al., 2023	Vitamin D deficiency enhances vascular oxidative stress, inflammation, and angiotensin II levels in the microcirculation of diabetic patients	No	irrelevant
35.	Quan et al., 2023	Impact of ultraviolet radiation on cardiovascular and metabolic disorders: The role of nitric oxide and vitamin D	No	irrelevant
36.	Yan et al., 2023	Combination therapy with saxagliptin and vitamin D for the preservation of β-cell function in adult-onset type 1 diabetes: a multi-center, randomized, controlled trial	No	different outcomes
37.	Sharp et al., 2023	Remission of Type II Diabetes Mellitus after Duodenal Switch: the Contribution of Common Channel Length	No	irrelevant

38.	Ahmed et al., 2023	Genetic scores associated with favourable and unfavourable adiposity have consistent effect on metabolic profile and disease risk across diverse ethnic groups	No	irrelevant
39.	Zhang et al., 2023	Association between serum 25- hydroxyvitamin D and albuminuria in middle-aged and older Chinese patients with type 2 diabetes	No	irrelevant
40.	Han et al., 2022	Analysis of risk factors for the development of type 2 diabetes mellitus complicated with Hashimoto’s thyroiditis	No	irrelevant
41.	Marcus et al., 2022	Pediatric obesity—Long-term consequences and effect of weight loss	No	irrelevant
42.	Al Masri et al., 2022	Nutritional and health status of adult Syrian refugees in the early years of asylum in Germany: a cross-sectional pilot study	No	irrelevant
43.	Šebunova et al., 2022	Changes in adipokine levels and metabolic profiles following bariatric surgery	No	different outcomes
44.	Tallon et al., 2022	Impact of diabetes status and related factors on COVID-19-associated hospitalization: A nationwide retrospective cohort study of 116,370 adults with SARS-CoV-2 infection	No	irrelevant
45.	Todorova et al., 2022	Vitamin D Status in a Bulgarian Population With Type 2 Diabetes and Diabetic Foot Ulcers	No	different outcomes
46.	Wang et al., 2022	Uygur type 2 diabetes patient fecal microbiota transplantation disrupts blood glucose and bile acid levels by changing the ability of the intestinal flora to metabolize bile acids in C57BL/6 mice	No	irrelevant
47.	Akbari et al., 2022	Comparison of serum levels of vitamin D in periodontitis patients with and without type 2 diabetes and healthy subjects	No	different outcomes
48.	Yavuz et al., 2022	Vitamin D receptor and estrogen receptor gene polymorphisms in men with type 2 diabetes: Effects on Bone Metabolism	No	different outcomes
49.	Maghbooli et al., 2022	Alterations in circulating levels of vitamin D binding protein, total and bioavailability of vitamin D in diabetic retinopathy patients	No	different outcomes
50.	Fu et al., 2022	Chinese patients with type 2 diabetes mellitus and nonalcoholic fatty liver disease have lower serum osteocalcin levels	No	different outcomes

		compared to individuals with type 2 diabetes mellitus and no liver disease		
51.	Mahmoodi et al., 2022	Associations between serum vitamin D3, atherogenic indices of plasma and cardiometabolic biomarkers among patients with diabetes in the KERCADR study	No	different outcomes
52.	De Sanctis et al., 2022	Insulin-like growth factor-1 (IGF-1) and glucose dysregulation in young adult patients with β-thalassemia major: causality or potential link?	No	irrelevant
53.	An et al., 2022	Micronutrient Supplementation to Reduce Cardiovascular Risk	No	different outcomes
54.	Chai et al., 2022	The effect of vitamin D supplementation on glycemic status and C-reactive protein levels in type 2 diabetic patients with ischemic heart disease: A protocol for systematic review and meta-analysis	No	irrelevant
55.	Zomorodian et al., 2022	Assessment of the relationship between 25-hydroxyvitamin D and albuminuria in type 2 diabetes mellitus	No	different outcomes
56.	Kibirige et al., 2022	Clinical, metabolic, and immunological characterisation of adult Ugandan patients with new-onset diabetes and low vitamin D status	No	different outcomes
57.	Hoseini et al., 2022	Decreased inflammatory gene expression accompanies the improvement of liver enzyme and lipid profile following aerobic training and vitamin D supplementation in T2DM patients	No	irrelevant
58.	Hoseini et al., 2022	Concurrent alteration in inflammatory biomarker gene expression and oxidative stress: how aerobic training and vitamin D improve T2DM	No	irrelevant
59.	Choudhury et al., 2022	Polycystic ovary syndrome (PCOS) increases the risk of subsequent gestational diabetes mellitus (GDM): A novel therapeutic perspective	No	irrelevant
60.	Maulood et al., 2021	Estimation of vitamin D receptor gene polymorphism in Type 2 Diabetes Mellitus patients in Erbil city	No	irrelevant
61.	Walters et al., 2021	Interrelationship between micronutrients and cardiovascular structure and function in type 2 diabetes	No	different outcomes

62.	Al-Daghri et al., 2021	Vitamin D status of Arab Gulf residents screened for SARS-CoV-2 and its association with COVID-19 infection	No	irrelevant
63.	Ma et al., 2021	Low 25-hydroxyvitamin D is associated with arterial stiffness in Chinese with Type 2 diabetes mellitus	No	different outcomes
64.	Mohd Yusof et al., 2021	Changes in dietary intake improve glycemic control following a structured nutrition therapy during Ramadan in individuals with type 2 diabetes	No	irrelevant
65.	Barham et al., 2021	The combination of omega−3 fatty acids with high doses of vitamin D3 elevate A1c levels: A randomized Clinical Trial in people with vitamin D deficiency	No	irrelevant
66.	Buchmann et al., 2021	Vitamin D insufficiency is associated with metabolic syndrome independent of insulin resistance and obesity in young adults	No	different outcomes
67.	Zhao et al., 2021	The relationship between vitamin D status and islet function in patients with type 2 diabetes mellitus	No	irrelevant
68.	Bejar et al., 2021	A Bidirectional Mendelian Randomization Study to evaluate the causal role of reduced blood vitamin D levels with type 2 diabetes risk in South Asians and Europeans	No	different outcomes
69.	Mittal et al., 2021	High prevalence of post COVID-19 fatigue in patients with type 2 diabetes: A case-control study	No	irrelevant
70.	Zaghloul et al., 2020	Clinical and metabolic characteristics of the Diabetes Intervention Accentuating Diet and Enhancing Metabolism (DIADEM-I) randomised clinical trial cohort	No	different outcomes
71.	Tay et al., 2020	Nutritional adequacy of very low- and high-carbohydrate, low saturated fat diets in adults with type 2 diabetes: A secondary analysis of a 2-year randomised controlled trial	No	different outcomes
72.	Pittas et al., 2020	Vitamin D supplementation for prevention of type 2 diabetes mellitus: To D or Not to D?	No	irrelevant
73.	Simon et al., 2020	Lifestyle and Environmental Approaches for the Primary Prevention of Hepatocellular Carcinoma	No	irrelevant
74.	Dawson-Hughes et al., 2020	Intratrial exposure to vitamin D and new-onset diabetes among adults with prediabetes: A secondary analysis from the vitamin D and type 2 diabetes (d2d) study	No	irrelevant

75.	Ding et al., 2020	Association between serum vitamin d metabolites and metabolic function in healthy asian adults	No	irrelevant
76.	Yonel et al., 2020	Patient acceptability of targeted risk-based detection of non-communicable diseases in a dental and pharmacy setting	No	irrelevant
77.	Brandi et al., 2020	Sexual Dimorphism of Coronavirus 19 Morbidity and Lethality	No	irrelevant
78.	Ahmed et al., 2020	Vitamin D3 metabolite ratio as an indicator of vitamin D status and its association with diabetes complications	No	irrelevant
79.	Gong et al., 2020	The relationship between serum vitamin D levels and lipid profile in hospitalized type 2 diabetic patients with different body weights	No	different outcomes
80.	Charoenngam et al., 2019	The ongoing D-lemma of Vitamin D supplementation for nonskeletal health and bone health	No	different outcomes
81.	Scanlon et al., 2019	Identification of Surrogate Biomarkers for the Prediction of Patients at Risk of Low Macular Pigment in Type 2 Diabetes	No	irrelevant
82.	Gröber et al., 2019	Diabetes prevention: Vitamin D supplementation may not provide any protection if there is no evidence of deficiency!	No	irrelevant
83.	Rui-hua et al., 2019	Decreased Levels of Serum IGF-1 and Vitamin D Are Associated With Cognitive Impairment in Patients With Type 2 Diabetes	No	different outcomes
84.	Ramos-Lopez et al., 2019	Interactions between drd2/ankk1 taqia polymorphism and dietary factors influence plasma triglyceride concentrations in diabetic patients from western mexico: A cross-sectional study	No	different outcomes
85.	Sadie-Van Gijsen et al., 2019	The Regulation of Marrow Fat by Vitamin D: Molecular Mechanisms and Clinical Implications	No	irrelevant
86.	Liu et al., 2019	Management of fracture risk in patients with diabetes—Chinese Expert Consensus	No	irrelevant
87.	Khan et al., 2019	Association of VDR gene variant (rs1544410) with type 2 diabetes in a Pakistani cohort	No	different outcomes
88.	Joseph et al., 2019	Hypoparathyroidism, Sensorineural deafness and renal disease (Barakat syndrome) caused by a reduced gene dosage in GATA3: A case report and review of literature	No	irrelevant

89.	Schmidt et al., 2019	Influence of Patient Setting and Dedicated Limb Salvage Efforts on Outcomes in Charcot-Related Foot Ulcer	No	irrelevant
90.	Ueda et al., 2019	Type 2 diabetes mellitus complicated with idiopathic hypoparathyroidism where poor glycaemic control was associated with low adherence to exercise and medication due to hypocalcaemia	No	different outcomes
91.	Yuan et al., 2019	Genetic prediction of serum 25-hydroxyvitamin D, calcium, and parathyroid hormone levels in relation to development of type 2 diabetes: A mendelian randomization study	No	different outcomes
92.	Rajabi-Naeeni et al., 2019	The effect of co supplementation of omega-3 and vitamin D on cardio metabolic risk factors and psychological distress in reproductive-aged women with prediabetes and hypovitaminosis D: A study protocol for a randomized controlled trial	No	irrelevant
93.	Hong et al., 2019	Randomized control trial comparing the effect of cilostazol and aspirin on changes in carotid intima-medial thickness	No	irrelevant
94.	Kim et al., 2019	The Differential Role of Vitamin D in Type 2 Diabetes Management and Control in Minority Populations	No	irrelevant
95.	De Boer et al., 2019	Effect of vitamin D and Omega-3 fatty acid supplementation on kidney function in patients with type 2 diabetes: A randomized clinical trial	No	different outcomes
96.	Zhang et al., 2019	The relationship between serum 25-hydroxyvitamin D concentration and type 2 diabetic peripheral neuropathy: A systematic review and a meta-analysis	No	different outcomes
97.	Wang et al., 2019	Relationship between serum 25-hydroxyvitamin D3 levels and severity of chronic periodontitis in type 2 diabetic patients: A cross-sectional study	No	different outcomes
98.	Goswami et al., 2019	Diabetes mellitus, Vitamin D & osteoporosis: Insights	No	different outcomes
99.	Al-Hazmi et al., 2019	Association of vitamin D deficiency and vitamin D receptor gene polymorphisms with type 2 diabetes mellitus saudi patients	No	different outcomes
100.	Vilarrasa et al., 2018	Nutritional deficiencies and bone metabolism after endobarrier in obese type 2 patients with diabetes	No	different outcomes

101.	Bener et al., 2018	Effect of ramadan fasting on glycemic control and other essential variables in diabetic patients	No	different outcomes
102.	Omar et al., 2018	Serum Vitamin D and Its Upregulated Protein, Thioredoxin Interacting Protein, Are Associated With Beta-Cell Dysfunction in Adult Patients With Type 1 and Type 2 Diabetes	No	irrelevant
103.	Patil et al., 2018	A clinical, biochemical profile of type-2 diabetes in women witspecial reference to vitamin-D status in obese and non-obese	No	irrelevant
104.	LeBlanc et al., 2018	Baseline characteristics of the Vitamin D and type 2 diabetes (D2d) study: A contemporary prediabetes cohort that will inform diabetes prevention efforts	No	different outcomes
105.	Funderburk et al., 2018	Prevalence of metabolic disease and correlation to body composition and cardiovascular fitness in adults undergoing fitness assessments	No	irrelevant
106.	Sezgin et al., 2018	Compliance with pathology testing guidelines in Australian general practice: Protocol for a secondary analysis of electronic health record data	No	irrelevant
107.	Angel et al., 2018	The association of VDR polymorphisms and type 2 diabetes in older people living in community in Santiago de Chile	No	different outcomes
108.	Nordklint et al., 2018	The effect of metformin versus placebo in combination with insulin analogues on bone mineral density and trabecular bone score in patients with type 2 diabetes mellitus	No	different outcomes
109.	Bo et al., 2018	Effects of resveratrol on bone health in type 2 diabetic patients. A double-blind randomized-controlled trial	No	different outcomes
110.	de Boer et al., 2018	Vitamin D and omega-3 trial to prevent and treat diabetic kidney disease: Rationale, design, and baseline characteristics	No	different outcomes
111.	Martínez-Laguna et al., 2018	Fracture risk in type 2 diabetic patients: A clinical prediction tool based on a large population-based cohort	No	irrelevant
112.	Rafiq et al., 2018	Body mass index, vitamin d, and type 2 diabetes: A systematic review and meta-analysis	No	irrelevant
113.	Cottam et al., 2018	An Analysis of Mid-Term Complications, Weight Loss, and Type 2 Diabetes Resolution of Stomach Intestinal Pylorus-	No	different outcomes

		Sparing Surgery (SIPS) Versus Roux-En-Y Gastric Bypass (RYGB) with Three-Year Follow-Up		
114.	Arslan et al., 2018	Early Results of Laparoscopic Sleeve Gastrectomy with Loop Bipartition	No	irrelevant
115.	Nakashima et al., 2018	Association between resistin and fibroblast growth factor 23 in patients with type 2 diabetes mellitus	No	different outcomes
116.	Reinehr et al., 2018	Vitamin D supplementation beyond the second year of life: Joint statement of the Nutrition Committee of the German Society for Pediatric and Adolescent Medicine and the German Society for Pediatric Endocrinology and Diabetology	No	irrelevant
117.	Tang et al., 2018	1,25-dihydroxyvitamin-D3 promotes neutrophil apoptosis in periodontitis with type 2 diabetes mellitus patients via the p38/MAPK pathway	No	different outcomes
118.	Ezhilarasi et al., 2018	BSMI single nucleotide polymorphism in vitamin D receptor gene is associated with decreased circulatory levels of serum 25-hydroxyvitamin D among micro and macrovascular complications of type 2 diabetes mellitus	No	different outcomes
119.	Zhong et al., 2018	Microangiopathy is associated with bone loss in female type 2 diabetes mellitus patients	No	different outcomes
120.	Milagres et al., 2017	Vitamin D insufficiency/deficiency is associated with insulin resistance in Brazilian children, regardless of body fat distribution	No	irrelevant
121.	Guo et al., 2017	Effects of serum 25-hydroxyvitaminD level on decreased bone mineral density at femoral neck and total hip in Chinese type 2 diabetes	No	different outcomes
122.	Glintborg et al., 2017	Medical comorbidity in polycystic ovary syndrome with special focus on cardiometabolic, autoimmune, hepatic and cancer diseases: An updated review	No	irrelevant
123.	Lessing et al., 2017	Laparoscopic sleeve gastrectomy for diabetics – 5-year outcomes	No	different outcomes

124.	de Waard et al., 2017	Optimal nutrition in lactating women and its effect on later health of offspring: A systematic review of current evidence and recommendations (EarlyNutrition project)	No	irrelevant
125.	Bentes et al., 2017	Association between muscle function and body composition, vitamin D status, and blood glucose in postmenopausal women with type 2 diabetes	No	irrelevant
126.	Singla et al., 2017	Vitamin D supplementation improves simvastatin-mediated decline in exercise performance: A randomized double-blind placebo-controlled study	No	different outcomes
127.	Bener et al., 2017	Interaction between diabetes mellitus and hypertension on risk of hearing loss in highly endogamous population	No	irrelevant
128.	Iqbal et al., 2017	Association of vitamin D binding protein polymorphism with risk of type 2 diabetes mellitus in a Pakistani urban population: A case control study	No	different outcomes
129.	Wang et al., 2017	Association between low serum 25-hydroxyvitamin D and depression in a large sample of Chinese patients with type 2 diabetes mellitus	No	different outcomes
130.	Bashir et al., 2017	Pattern Of Dyslipidaemia And Its Association With Hypovitaminosis D In Type 2 Diabetes Mellitus	No	different outcomes
131.	Liyanage et al., 2017	Effects of high-dose parenteral vitamin D therapy on lipid profile and blood pressure in patients with diabetic nephropathy: A randomized double-blind clinical trial	No	different outcomes
132.	Rahman et al., 2017	Association of vitamin D and vitamin D binding protein (DBP) gene polymorphism with susceptibility of type 2 diabetes mellitus in Bangladesh	No	different outcomes
133.	Nunez Lopez et al., 2017	Circulating levels of miR-7, miR-152 and miR-192 respond to vitamin D supplementation in adults with prediabetes and correlate with improvements in glycemic control	No	irrelevant
134.	Silva et al., 2017	Klotho levels: association with insulin resistance and albumin-to-creatinine ratio in type 2 diabetic patients	No	irrelevant

135.	Machytka et al., 2017	Partial jejunal diversion using an incisionless magnetic anastomosis system: 1-year interim results in patients with obesity and diabetes	No	different outcomes
136.	Bertoccini et al., 2017	The vitamin D receptor functional variant rs2228570 (C>T) does not associate with type 2 diabetes mellitus	No	different outcomes
137.	Cimini et al., 2017	Circulating IL-8 levels are increased in patients with type 2 diabetes and associated with worse inflammatory and cardiometabolic profile	No	irrelevant
138.	Arsenault et al., 2016	Emerging Cardiovascular Disease Biomarkers and Incident Diabetes Mellitus Risk in Statin-Treated Patients With Coronary Artery Disease	No	different outcomes
139.	Miller et al., 2016	Evaluation of Quantitative Computed Tomography Cortical Hip Quadrant in a Clinical Trial With Rosiglitazone: A Potential New Study Endpoint	No	different outcomes
140.	Caretta et al., 2016	Hypovitaminosis D is associated with erectile dysfunction in type 2 diabetes	No	different outcomes
141.	Wagenknecht et al., 2016	Bone mineral density and progression of subclinical atherosclerosis in African-Americans with type 2 diabetes	No	different outcomes
142.	Gilhotra et al., 2016	Non-traumatic lower limb amputation in patients with end-stage renal failure on dialysis: an Australian perspective	No	irrelevant
143.	Smetana et al., 2016	Should we screen for type 2 diabetes? Grand rounds discussion from Beth Israel Deaconess Medical Center	No	irrelevant
144.	Einbinder et al., 2016	Glucagon-like peptide-1 and vitamin D: anti-inflammatory response in diabetic kidney disease in db/db mice and in cultured endothelial cells	No	irrelevant
145.	Calvo et al., 2016	A Retrospective Study in Adults with Metabolic Syndrome: Diabetic Risk Factor Response to Daily Consumption of Agaricus bisporus (White Button Mushrooms)	No	different outcomes
146.	Via et al., 2016	Nutrition in Type 2 Diabetes and the Metabolic Syndrome	No	irrelevant

147.	Barchetta et al., 2016	Dipeptidyl peptidase-4 inhibitors and bone metabolism: is vitamin D the link?	No	irrelevant
148.	Bener et al., 2016	The role of vitamin D, obesity and physical exercise in regulation of glycemia in Type 2 Diabetes Mellitus patients	No	irrelevant
149.	Gangloff et al., 2016	Changes in circulating Vitamin D levels with loss of adipose tissue	No	different outcomes
150.	Rana et al., 2016	A relationship between vitamin D, parathyroid hormone, calcium levels and lactose intolerance in type 2 diabetic patients and healthy subjects	No	different outcomes
151.	Yu et al., 2016	The genetic polymorphisms in vitamin D receptor and the risk of type 2 diabetes mellitus: an updated meta-analysis	No	different outcomes
152.	Sergeev et al., 2016	Vitamin D—Cellular Ca2+ link to obesity and diabetes	No	different outcomes
153.	Ströhle et al., 2016	Health outcomes of vegetarian nutrition – An update	No	irrelevant
154.	Fleury et al., 2016	Sun exposure and its effects on human health: Mechanisms through which sun exposure could reduce the risk of developing obesity and cardiometabolic dysfunction	No	irrelevant
155.	Yi et al., 2016	Vitamin D receptor down-regulation is associated with severity of albuminuria in type 2 diabetes patients	No	different outcomes
156.	Scott et al., 2016	Sarcopenia: A potential cause and consequence of type 2 diabetes in Australia's ageing population?	No	irrelevant
157.	Langer et al., 2016	Insufficient Vitamin D Response to Solar Radiation in German Patients with Type 2 Diabetes or Gestational Diabetes	No	different outcomes
158.	Billeter et al., 2015	Risk of Malnutrition, Trace Metal, and Vitamin Deficiency Post Roux-en-Y Gastric Bypass—a Prospective Study of 20 Patients with BMI <35 kg/m2	No	different outcomes
159.	Moore et al., 2015	Beta Cell Function and the Nutritional State: Dietary Factors that Influence Insulin Secretion	No	irrelevant

160.	Pham et al., 2015	The relationship of serum 25-hydroxyvitamin D and insulin resistance among nondiabetic Canadians: A longitudinal analysis of participants of a preventive health program	No	different outcomes
161.	Zostautiene et al., 2015	Genetic variations in the Vitamin D receptor predict type 2 diabetes and myocardial infarction in a community-based population: The tromsø study	No	different outcomes
162.	Zagami et al., 2015	Low circulating vitamin D levels are associated with increased arterial stiffness in prediabetic subjects identified according to HbA1c	No	different outcomes
163.	Wamberg et al., 2015	Causes of Vitamin D Deficiency and Effect of Vitamin D Supplementation on Metabolic Complications in Obesity: a Review	No	irrelevant
164.	Vieira et al., 2015	Falls, physical limitations, confusion and memory problems in people with type II diabetes, undiagnosed diabetes and prediabetes, and the influence of vitamins A, D and E	No	irrelevant
165.	Al-Shahwan et al., 2015	Effects of 12-month, 2000IU/day vitamin D supplementation on treatment naïve and vitamin D deficient Saudi type 2 diabetic patients	No	irrelevant
166.	Calvo-Romero et al., 2015	Vitamin D levels in patients with type 2 diabetes mellitus	No	irrelevant
167.	Jia et al., 2015	Vitamin D Receptor Genetic Polymorphism Is Significantly Associated with Risk of Type 2 Diabetes Mellitus in Chinese Han Population	No	different outcomes
168.	Maghrabi et al., 2015	Two-year outcomes on bone density and fracture incidence in patients with T2DM randomized to bariatric surgery versus intensive medical therapy	No	different outcomes
169.	Shoukry et al., 2015	Urinary monocyte chemoattractant protein-1 and vitamin D-binding protein as biomarkers for early detection of diabetic nephropathy in type 2 diabetes mellitus	No	different outcomes
170.	Caretta et al., 2015	Hypovitaminosis D is associated with lower urinary tract symptoms and benign prostate hyperplasia in type 2 diabetes	No	different outcomes
171.	Hart et al., 2015	Prevalence, risk factors and sequelae of Staphylococcus aureus carriage in diabetes: The Fremantle Diabetes Study Phase II	No	irrelevant

172.	Ibrahim et al., 2015	Study of the effect of vitamin D supplementation on glycemic control in type 2 diabetic prevalent hemodialysis patients	No	irrelevant
173.	Yang et al., 2015	Anti-inflammatory effects of 1,25-dihydroxyvitamin D3 in monocytes cultured in serum from patients with type 2 diabetes mellitus and diabetic nephropathy with uremia via Toll-like receptor 4 and nuclear factor-B p65	No	irrelevant
174.	Freedman et al., 2015	Vitamin D associations with renal, bone, and cardiovascular phenotypes: African American-diabetes heart study	No	different outcomes
175.	Perez-Diaz et al., 2015	The impact of Vitamin D levels on glycemic control and bone mineral density in postmenopausal women with type 2 diabetes	No	irrelevant
176.	Freedman et al., 2015	Plasma FGF23 and Calcified Atherosclerotic Plaque in African Americans with Type 2 Diabetes Mellitus	No	different outcomes
177.	Baldwin et al., 2014	The Primary Care Physician/Nephrologist Partnership in Treating Chronic Kidney Disease	No	irrelevant
178.	Pittas et al., 2014	Rationale and design of the vitamin D and type 2 diabetes (D2d) study: A diabetes prevention trial	No	different outcomes
179.	Tabesh et al., 2014	Calcium-vitamin D cosupplementation influences circulating inflammatory biomarkers and adipocytokines in vitamin D-insufficient diabetics: A randomized controlled clinical trial	No	irrelevant
180.	Dutta et al., 2014	Serum fetuin-A concentration predicts glycaemic outcomes in people with prediabetes: A prospective study from eastern India	No	different outcomes
181.	Bakalov et al., 2014	Lack of correlation of the serum 25(OH) vitamin D levels with the glycated hemoglobin A1c and the lipid profile in type 2 diabetes patients on oral antidiabetic drugs - Preliminary data	No	irrelevant
182.	Elkassaby et al., 2014	A randomised controlled trial of high dose vitamin D in recent-onset type 2 diabetes	No	irrelevant
183.	Al-Zahrani et al., 2014	A 3-month oral vitamin D supplementation marginally improves diastolic blood pressure in Saudi patients with type 2 diabetes mellitus	No	different outcomes
184.	He et al., 2014	Vitamin D deficiency increases the risk of retinopathy in Chinese patients with Type 2 diabetes	No	different outcomes

185.	Daly et al., 2014	The effects of progressive resistance training combined with a whey-protein drink and vitamin D supplementation on glycaemic control, body composition and cardiometabolic risk factors in older adults with type 2 diabetes: Study protocol for a randomized controlled trial	No	different outcomes
186.	Kiskac et al., 2014	Evaluation of the relationship between serum apelin levels and vitamin D and mean platelet volume in diabetic patients	No	irrelevant
187.	Muñoz-Prieto et al., 2021	Untargeted metabolomic profiling of serum in dogs with hypothyroidism	No	irrelevant
188.	Briguglio et al., 2020	Vitamin D, cardio-inflammation, and endothelial dysfunction in older adults after orthopedic surgery: Results from an open-label trial to ameliorate cardiac function	No	different outcomes
189.	Lichtenstein et al., 2013	Vitamin D: non-skeletal actions and rational use	No	irrelevant
190.	Weiler et al., 2013	Osteocalcin and vitamin D status are inversely associated with homeostatic model assessment of insulin resistance in Canadian Aboriginal and white women	No	irrelevant
191.	Wu et al., 2017	Vitamin D supplementation and glycemic control in type 2 diabetes patients: A systematic review and meta-analysis	No	irrelevant
192.	Damsgaard et al., 2020	Can vitamin D supplementation improve childhood cardiometabolic status?—data from 2 randomized trials	No	Irrelevant (population: children)
193.	Della Pepa et al., 2020	Effects of a multifactorial ecosustainable isocaloric diet on liver fat in patients with type 2 diabetes: randomized clinical trial	No	Different outcomes
194.	Nikooyeh et al., 2021	Daily intake of yogurt drink fortified either with vitamin D alone or in combination with added calcium causes a thyroid-independent increase of resting metabolic rate in adults with type 2 diabetes	No	Different outcomes
195.	Mudjanarko et al., 2022	Effects of aerobic exercise on adiponectin levels potentially mediated by vitamin D in type 2 diabetic patients	No	Different outcomes
196.	Zhou et al., 2014	Effects of vitamin D supplementation on insulin resistance in patients with type 2 diabetes mellitus	No	irrelevant

197.	Karalliedde et al., 2023	Effect of calcitriol treatment on arterial stiffness in people with type 2 diabetes and stage 3 chronic kidney disease	No	Different outcomes
198.	Yi et al., 2016	Vitamin D receptor down-regulation is associated with severity of albuminuria in type 2 diabetes patients	No	Different outcomes
199.	Miller et al., 2021	Effects of whey protein plus vitamin D supplementation combined with progressive resistance training on glycaemic control…	No	irrelevant
200.	Kawahara et al., 2022	Effect of active vitamin D treatment on development of type 2 diabetes: DPVD randomised controlled trial in Japanese population	No	irrelevant
201.	Shab-Bidar et al., 2015	Vitamin D receptor Cdx-2-dependent response of central obesity to vitamin D intake in subjects with type 2 diabetes	No	Different outcomes
202.	Mirzavandi et al., 2020	The effect of intramuscular megadose of vitamin D injections on E-selectin, CRP and biochemical parameters…	No	irrelevant
203.	Vrzhesinskaya et al., 2022	Vitamin supply of patients with type 2 diabetes mellitus complicated by nephropathy	No	Different outcomes
204.	Penckofer et al., 2022	Vitamin D Supplementation for the Treatment of Depressive Symptoms in Women with Type 2 Diabetes	No	Different outcomes
205.	Johny et al., 2022	Vitamin D Supplementation Modulates Platelet-Mediated Inflammation in Subjects With Type 2 Diabetes	No	irrelevant
206.	Nordklint et al., 2021	Effect of metformin and insulin vs. placebo and insulin on whole body composition in overweight patients with type 2 diabetes	No	Different outcomes
207.	Cardoso et al., 2020	Bone mineral density in severely obese women: health risk and health protective risk factors in three different bone sites	No	Different outcomes
208.	Mahmoodi et al., 2022	Associations between serum vitamin D3, atherogenic indices of plasma and cardiometabolic biomarkers among patients with diabetes	No	Different outcomes
209.	Maggi et al., 2014	Vitamin D deficiency, serum leptin and osteoprotegerin levels in older diabetic patients	No	Different outcomes
210.	Ehrampoush et al., 2021	The association of vitamin D levels and insulin resistance	No	-

211.	Mohd Yusof et al., 2021	Changes in dietary intake improve glycemic control following structured nutrition therapy during Ramadan	No	Different outcomes
212.	Dawson-Hughes et al., 2020	Intratrial Exposure to Vitamin D and New-Onset Diabetes Among Adults With Prediabetes	No	irrelevant
213.	Hsia et al., 2023	Effect of Vitamin D on Regression to Normal Glucose Regulation and Individual Glycemic Measures	No	irrelevant
214.	Barchetta et al., 2016	No effects of oral vitamin D supplementation on non-alcoholic fatty liver disease in patients with type 2 diabetes	No	different outcomes
215.	Koch et al., 2017	Vitamin D supplementation enhances C18(Dihydro)ceramide levels in type 2 diabetes patients	No	different outcomes
216.	Strobel et al., 2014	Effect of a randomised controlled vitamin D trial on insulin resistance and glucose metabolism in patients with type 2 diabetes mellitus	No	irrelevant
217.	Gariballa et al., 2022	Vitamin D [25(OH)D] metabolites and epimers in obese subject: interaction and correlations with adverse metabolic health risk factors	No	different outcomes
218.	Neyestani et al., 2015	A Vitamin D-Calcium-Fortified Yogurt Drink Decreased Serum PTH but did not Affect Osteocalcin in Subjects with Type 2 Diabetes	No	different outcomes
219.	Derosa et al., 2022	Vitamin D3 supplementation improves glycemic control in type 2 diabetic patients	No	irrelevant
220.	Angellotti et al., 2019	Effect of vitamin D supplementation on cardiovascular risk in type 2 diabetes	No	different outcomes
221.	Memelink et al., 2020	Effect of an Enriched Protein Drink on Muscle Mass and Glycemic Control during Combined Lifestyle Intervention in Older Adults with Obesity and Type 2 Diabetes	No	different outcomes
222.	Yuan et al., 2019	Genetic Prediction of Serum 25-Hydroxyvitamin D, Calcium, and Parathyroid Hormone Levels in Relation to Development of Type 2 Diabetes	No	different outcomes
223.	Nikooyeh et al., 2014	Daily intake of vitamin D- or calcium-vitamin D-fortified Persian yogurt drink attenuates diabetes-induced oxidative stress	No	different outcomes

224.	Behshad et al., 2022	The short-term effect of high dose vitamin D3 supplementation in improving hypovitaminosis in patients with type 2 diabetes	No	irrelevant
225.	Kim et al., 2021	No effect of vitamin D supplementation on metabolic parameters but on lipids in patients with type 2 diabetes and chronic kidney disease	No	different outcomes
226.	Khan et al., 2018	Efficacy of oral vitamin D on glycated haemoglobin (HbA1c) in type 2 diabetics having vitamin D deficiency	No	irrelevant
227.	Dwivedi et al., 2017	Parenteral vitamin D supplementation is superior to oral in vitamin D insufficient patients with type 2 diabetes mellitus	No	irrelevant
228.	Shab-Bidar et al., 2015	The interactive effect of improvement of vitamin D status and VDR FokI variants on oxidative stress in type 2 diabetic subjects	No	different outcomes
229.	Shehab et al., 2015	Effect of short-term oral vitamin D supplementation on peripheral neuropathy in type 2 diabetes mellitus	No	different outcomes
230.	McCarthy et al., 2017	Nurse Coaching vs. Herbal Supplementation for Weight Reduction in Soldiers	No	irrelevant
231.	Virtanen et al., 2025	Effect of vitamin D3 supplementation on incidence of type 2 diabetes in healthy older adults	No	irrelevant population
232.	Swart et al., 2018	Effects of Vitamin D supplementation on markers for cardiovascular disease and type 2 diabetes	No	irrelevant
233.	Gnudi et al., 2023	Effect of active vitamin-D on left ventricular mass index in type 2 diabetes and chronic kidney disease	No	different outcomes
234.	Koch et al., 2017	Vitamin D supplementation enhances C18(Dihydro)ceramide levels in type 2 diabetes patients	No	different outcomes
235.	Strobel et al., 2014	Effect of a randomised controlled vitamin D trial on insulin resistance and glucose metabolism in patients with type 2 diabetes mellitus	No	irrelevant
236.	Gariballa et al., 2022	Vitamin D [25(OH)D] metabolites and epimers in obese subject: interaction and correlations with adverse metabolic health risk factors	No	different outcomes

237.	Neyestani et al., 2015	A Vitamin D-Calcium-Fortified Yogurt Drink Decreased Serum PTH but did not Affect Osteocalcin in Subjects with Type 2 Diabetes	No	different outcomes
238.	Derosa et al., 2022	Vitamin D3 supplementation improves glycemic control in type 2 diabetic patients	No	irrelevant
239.	Angellotti et al., 2019	Effect of vitamin D supplementation on cardiovascular risk in type 2 diabetes	No	different outcomes
240.	Memelink et al., 2020	Effect of an Enriched Protein Drink on Muscle Mass and Glycemic Control during Combined Lifestyle Intervention in Older Adults with Obesity and Type 2 Diabetes	No	different outcomes
241.	Yuan et al., 2019	Genetic Prediction of Serum 25-Hydroxyvitamin D, Calcium, and Parathyroid Hormone Levels in Relation to Development of Type 2 Diabetes	No	different outcomes
242.	Shab-Bidar et al., 2015	The interactive effect of improvement of vitamin D status and VDR FokI variants on oxidative stress in type 2 diabetic subjects	No	different outcomes
243.	Shehab et al., 2015	Effect of short-term oral vitamin D supplementation on peripheral neuropathy in type 2 diabetes mellitus	No	different outcomes
244.	McCarthy et al., 2017	Nurse Coaching vs. Herbal Supplementation for Weight Reduction in Soldiers	No	irrelevant
245.	Virtanen et al., 2025	Effect of vitamin D3 supplementation on incidence of type 2 diabetes in healthy older adults	No	irrelevant population
246.	Swart et al., 2018	Effects of Vitamin D supplementation on markers for cardiovascular disease and type 2 diabetes	No	irrelevant
247.	Gnudi et al., 2023	Effect of active vitamin-D on left ventricular mass index in type 2 diabetes and chronic kidney disease	No	different outcomes
248.	Bener et al., 2016	The role of vitamin D, obesity and physical exercise in regulation of glycemia in Type 2 Diabetes Mellitus patients	No	different outcomes
249.	Gharib et al., 2022	Association of Vitamin D Deficiency, Dyslipidemia, and Obesity with the Incidence of Coronary Artery Diseases in Type 2 Diabetic Saudi Patients	No	different outcomes

250.	de Luis et al., 2021	The lactase rs4988235 is associated with obesity related variables and diabetes mellitus in menopausal obese females	No	irrelevant
251.	Mikolasevic et al., 2020	Screening for nonalcoholic fatty liver disease in patients with type 2 diabetes mellitus using transient elastography	No	different outcomes
252.	Nordklint et al., 2021	Effect of metformin and insulin vs. placebo and insulin on whole body composition in overweight patients with type 2 diabetes: a randomized placebo-controlled trial	No	different outcomes
253.	Dhas et al., 2021	Serum 25(OH)D Concentration and Cardiovascular Disease Risk Markers among Middle-Aged Healthy and Type 2 Diabetic Subjects	No	different outcomes
254.	Cătoi et al., 2021	Relationship between 25 hydroxyvitamin D, overweight/obesity status, pro-inflammatory and oxidative stress markers in patients with type 2 diabetes	No	different outcomes
255.	Abela et al., 2021	Why is the incidence of type 1 diabetes increasing?	No	irrelevant
256.	Duan et al., 2021	Gut microbiota as the critical correlation of polycystic ovary syndrome and type 2 diabetes mellitus	No	irrelevant
257.	Carberry et al., 2021	Mononeuropathy Multiplex after COVID-19	No	irrelevant
258.	Kim et al., 2021	No effect of vitamin D supplementation on metabolic parameters but on lipids in patients with type 2 diabetes and chronic kidney disease	No	different outcomes
259.	Ojanen et al., 2021	Towards early risk biomarkers: serum metabolic signature in childhood predicts cardio-metabolic risk in adulthood	No	irrelevant
260.	Volek et al., 2021	Alternative dietary patterns for Americans: Low-carbohydrate diets	No	irrelevant
261.	Selvarajan et al., 2021	Association of genetic polymorphisms in vitamin D receptor (ApaI, TaqI and FokI) with vitamin D and glycemic status in type 2 diabetes patients from Southern India	No	irrelevant
262.	Waage et al., 2023	Cohort profile update: the Norwegian STORK Groruddalen pregnancy and birth cohort—the role of ethnicity and causal pathways for obesity, type 2 diabetes, cardiovascular disease and other health issues	No	irrelevant

263.	Habiba et al., 2023	Effect of oral versus parenteral vitamin D3 supplementation on nuclear factor-κB and platelet aggregation in type 2 diabetic patients	No	different outcomes
264.	Nhan et al., 2023	Plant-based diets: a fad or the future of medical nutrition therapy for children with chronic kidney disease?	No	irrelevant
265.	Papatheodoridi et al., 2023	Health-related Quality of Life in Patients With Nonalcoholic Fatty Liver Disease: A Prospective Multi-center UK Study	No	different outcomes
266.	Volpe et al., 2023	The Emerging Role of Prediabetes and Its Management: Focus on l-Arginine and a Survey in Clinical Practice	No	irrelevant
267.	Taderegew et al., 2023	Vitamin D deficiency and its associated factors among patients with type 2 diabetes mellitus: a systematic review and meta-analysis	No	different outcomes
268.	Argano et al., 2023	The Role of Vitamin D and Its Molecular Bases in Insulin Resistance, Diabetes, Metabolic Syndrome, and Cardiovascular Disease: State of the Art	No	different outcomes
269.	Af Geijerstam et al., 2023	Smoking and cardiovascular disease in patients with type 2 diabetes: a prospective observational study	No	different outcomes
270.	Wang et al., 2023	Characteristics and Burdens of Disease in Patients from Beijing with Generalized Pustular Psoriasis and Palmoplantar Pustulosis: Multicenter Retrospective Cohort Study Using a Regional Database	No	irrelevant
271.	Mesinovic et al., 2023	Type 2 Diabetes Mellitus and Sarcopenia as Comorbid Chronic Diseases in Older Adults: Established and Emerging Treatments and Therapies	No	irrelevant
272.	Aroda et al., 2019	Establishing an electronic health record-supported approach for outreach to and recruitment of persons at high risk of type 2 diabetes in clinical trials: The vitamin D and type 2 diabetes (D2d) study experience	No	irrelevant
273.	Shehab et al., 2015	Prospective evaluation of the effect of short-term oral vitamin D supplementation on peripheral neuropathy in type 2 diabetes mellitus	No	different outcomes

274.	Ryu et al., 2014	The effect of high-dose vitamin D supplementation on insulin resistance and arterial stiffness in patients with type 2 diabetes	No	irrelevant
275.	Forouhi et al., 2016	Effects of vitamin D2 or D3 supplementation on glycaemic control and cardiometabolic risk among people at risk of type 2 diabetes	No	irrelevant
276.	LeBlanc et al., 2018	Baseline Characteristics of the Vitamin D and Type 2 Diabetes (D2d) Study: A Contemporary Prediabetes Cohort That Will Inform Diabetes Prevention Efforts	No	irrelevant
277.	de Paula et al., 2020	Efficacy of single-dose cholecalciferol in the blood pressure of patients with type 2 diabetes, hypertension and hypovitaminoses D	No	different outcomes
278.	Riek et al., 2018	Vitamin D3 supplementation decreases a unique circulating monocyte cholesterol pool in patients with type 2 diabetes	No	different outcomes
279.	Dalan et al., 2016	A randomised controlled trial evaluating the impact of targeted vitamin D supplementation on endothelial function in type 2 diabetes mellitus: The DIMENSION trial	No	different outcomes
280.	Krul-Poel et al., 2014	Study protocol: a randomised placebo-controlled clinical trial to study the effect of vitamin D supplementation on glycaemic control in type 2 Diabetes Mellitus SUNNY trial	No	different outcomes
281.	Gagnon et al., 2014	Effects of combined calcium and vitamin D supplementation on insulin secretion, insulin sensitivity and β-cell function in multi-ethnic vitamin D-deficient adults at risk for type 2 diabetes	No	irrelevant
282.	Sylvetsky et al., 2017	Pigment Epithelium-Derived Factor Declines in Response to an Oral Glucose Load and Is Correlated with Vitamin D and BMI but Not Diabetes Status in Children and Young Adults	No	irrelevant
283.	Alam et al., 2017	Improvement in Neuropathy Specific Quality of Life in Patients with Diabetes after Vitamin D Supplementation	No	different outcomes
284.	Mitchell et al., 2015	Insulin secretion and sensitivity in healthy adults with low vitamin D are not affected by high-dose ergocalciferol administration: a randomized controlled trial	No	irrelevant
