# Supplementary material for: The Impact of Vitamin D Supplementation on Fasting Plasma Glucose, Insulin Sensitivity, and Inflammation in Type 2 Diabetes Mellitus: A Systematic Review and Meta-Analysis
Source: Nutrients. 2025 Jul 30;17(15):2489. doi: 10.3390/nu17152489 (PMC12348612; doi:10.3390/nu17152489)
Supplement: Supplementary file 1 [file nutrients-17-02489-s001.zip › Supplementary File S3_Risk of Bias Assessment.pdf]

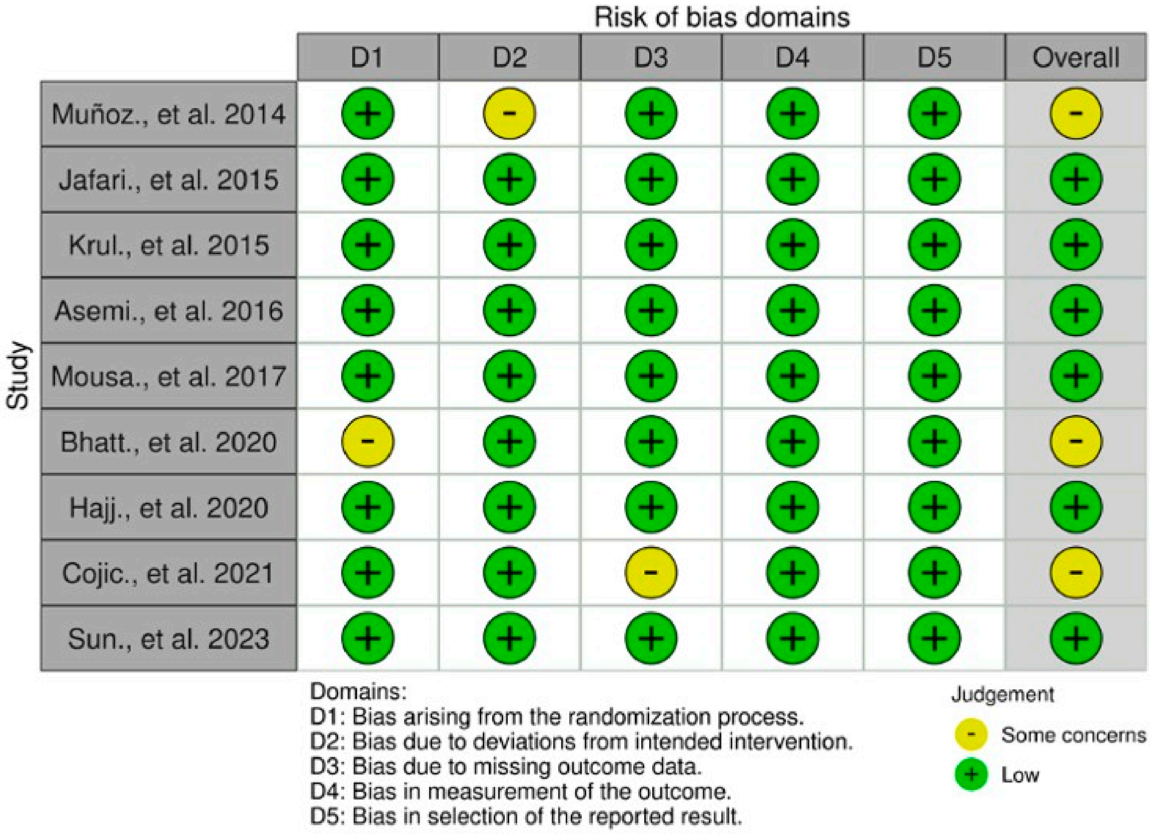

Risk of bias domains
D1
D2
D3
D4
D5
Overall
Study
Muñoz., et al. 2014
Jafari., et al. 2015
Krul., et al. 2015
Asemi., et al. 2016
Mousa., et al. 2017
Bhatt., et al. 2020
Hajj., et al. 2020
Cojic., et al. 2021
Sun., et al. 2023
Domains:
D1: Bias arising from the randomization process.
D2: Bias due to deviations from intended intervention.
D3: Bias due to missing outcome data.
D4: Bias in measurement of the outcome.
D5: Bias in selection of the reported result.
Judgement
Some concerns
Low

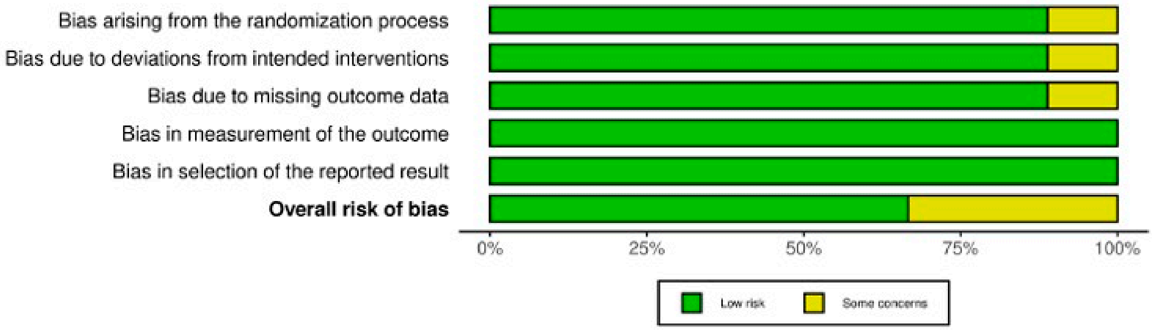

Bias arising from the randomization process
Bias due to deviations from intended interventions
Bias due to missing outcome data
Bias in measurement of the outcome
Bias in selection of the reported result
Overall risk of bias
0%
25%
50%
75%
100%
Low risk
Some concerns
